# *XPO1*^E571K^ Mutation Modifies Exportin 1 Localisation and Interactome in B-Cell Lymphoma

**DOI:** 10.3390/cancers12102829

**Published:** 2020-09-30

**Authors:** Hadjer Miloudi, Élodie Bohers, François Guillonneau, Antoine Taly, Vincent Cabaud Gibouin, Pierre-Julien Viailly, Gaëtan Jego, Luca Grumolato, Fabrice Jardin, Brigitte Sola

**Affiliations:** 1 INSERM U1245, Unicaen, Normandie University, F-14000 Caen, France; hadjer.miloudi@unicaen.fr (H.M.); elodie.bohers@chb.unicancer.fr (E.B.); pierre-julien.viailly@chb.unicancer.fr (P.-J.V.); fabrice.jardin@chb.unicancer.fr (F.J.); 2 Centre de lutte contre le Cancer Henri Becquerel, F-76000 Rouen, France; 3 Plateforme Protéomique 3P5, Université de Paris, Institut Cochin, INSERM, CNRS, F-75014 Paris, France; francois.guillonneau@parisdescartes.fr; 4 Laboratoire de Biochimie Théorique, CNRS UPR 9030, Université de Paris, F-75005 Paris, France; taly@ibpc.fr; 5 Institut de Biologie Physico-Chimique, Fondation Edmond de Rothschild, PSL Research University, F-75005 Paris, France; 6 INSERM, LNC UMR1231, F-21000 Dijon, France; vincent.cabaud-gibouin@u-bourgogne.fr (V.C.G.); gaetan.jego@u-bourgogne.fr (G.J.); 7 Team HSP-Pathies, University of Burgundy and Franche-Comtée, F-21000 Dijon, France; 8 INSERM U1239, Unirouen, Normandie University, F-76130 Mont-Saint-Aignan, France; luca.grumolato@univ-rouen.fr

**Keywords:** B-cell lymphoma, XPO1/CRM1, nuclear export, CRISPR–Cas9, proteomics, nuclear import, importin β1, indirect immunofluorescence, proximity ligation assay

## Abstract

**Simple Summary:**

Almost 25% of patients with either primary mediastinal B-cell lymphoma (PMBL) or classical Hodgkin lymphoma (cHL) possess a recurrent mutation of the *XPO1* gene encoding the major nuclear export protein. The aim of our study was to assess the molecular function of the mutant XPO1 protein. Using several cell models (including CRISPR–Cas9 edited cells) and high throughput techniques, we determined that the export capacity of the mutant XPO1 was not altered. However, mutant XPO1 accumulated in the cytoplasm due to its binding to importin β1 (or IPO1). The targeting of XPO1 is largely efficient for fighting haemopathies. The inhibition of IPO1 could open new therapeutic perspectives for B-cell lymphomas.

**Abstract:**

The *XPO1* gene encodes exportin 1 (XPO1) that controls the nuclear export of cargo proteins and RNAs. Almost 25% of primary mediastinal B-cell lymphoma (PMBL) and classical Hodgkin lymphoma (cHL) cases harboured a recurrent *XPO1* point mutation (NM_003400, chr2:g61718472C>T) resulting in the E571K substitution within the hydrophobic groove of the protein, the site of cargo binding. We investigated the impact of the *XPO1*^E571K^ mutation using PMBL/cHL cells having various *XPO1* statuses and CRISPR–Cas9-edited cells in which the E571K mutation was either introduced or knocked-out. We first confirmed that the mutation was present in both XPO1 mRNA and protein. We observed that the mutation did not modify the export capacity but rather the subcellular localisation of XPO1 itself. In particular, mutant XPO1 bound to importin β1 modified the nuclear export/import dynamics of relevant cargoes.

## 1. Introduction

Primary mediastinal B-cell lymphoma (PMBL) is an aggressive B-cell lymphoma clinically and molecularly distinct from germinal centre B-cell-like (GCB) and activated B-cell-like (ABC) subtypes of diffuse large B-cell lymphoma (DLBCL) [1,2]. PMBL cells are characterized by genomic abnormalities that are also found in the classical form of Hodgkin lymphoma (cHL) [3]. These observations underline the molecular relationships between these two haematological malignancies [2]. We previously described a recurrent mutation of the *XPO1* gene occurring with the same frequency in both PMBL and cHL (25%) [4,5]. This mutation appears as a genetic feature of these two types of lymphoma, since it is present at low frequency or absent in GCB or ABC lymphomas [6].

XPO1 (previously known as CRM1, chromosome region maintenance 1) is the major eukaryotic nuclear export protein. XPO1 mediates the translocation of several types of RNAs, ribonucleoprotein complexes and more than 200 cargoes, including tumour suppressors and regulatory proteins [7]. Overexpression, deregulation or dysfunction of XPO1 have been reported in various types of cancer [7]. In haematologic malignancies, quantitative (amplification of *XPO1* or translocation) and qualitative (*XPO1* mutation) abnormalities have been described. However, although XPO1 overexpression is observed in myeloid and lymphoid lineages, in both acute and chronic diseases, mutations have been described only for PMBL [4], cHL [5] and, with a lower frequency, in chronic lymphocytic leukaemia (CLL) [8,9] or DLBCL [4]. All reported *XPO1* mutations lead to a substitution of glutamate 571, most frequently to lysine. 

Using PMBL and cHL cell lines with various *XPO1* statuses and CRISPR–Cas9-edited cells, we investigated the effects of the *XPO1*^E571K^ mutation on XPO1 interactome. We first confirmed that the mutation was present at the mRNA and protein levels. Strikingly, comparing PMBL/cHL cell lines and edited clones in which we replaced one wild-type (wt) allele with a mutant one or deleted a mutant allele, we observed that the accumulation of XPO1 at the perinuclear rim correlated with the presence of the mutant protein. We analysed the interactomes of both wt and mutant proteins and found that most cargoes were similar. However, some XPO1 partners were specifically associated with the wt or mutant proteins, and among them, the karyopherin β1 (also known as importin β1, KPNB1 or IPO1) was specifically associated with the XPO1^E571K^ form. We further confirmed the XPO1/IPO1 interaction with the proximity-ligation assay (PLA) and by using importazole, an inhibitor of the IPO1 receptor. Our data uncovered one functional impact of the E571K mutation that could be involved in the B-cell lymphoma physiopathology.

## 2. Results

### 2.1. PMBL and cHL Parental and Edited Cell Lines Display Various XPO1 Status

Karpas 1106-P (hereafter referred to as K1106), MedB1 and U2940 PMBL cell lines and UH-01 cell line have been previously described [4,5]. The quantification of the mutant vs. wt alleles in PMBL/cHL cells was performed by genomic (g)DNA and RNA pyrosequencing (Tables S1 and S2). The percentages of the mutant and wt XPO1 forms were similar in MedB1 cells, indicating that the cell line is heterozygous for XPO1, whereas only the wt XPO1 allele was present in K1106 and U2940 cells. UH-01 cells exhibited a duplication of the mutant allele and one wt XPO1 allele (Table S1). XPO1 status was next confirmed by reverse transcriptase multiplex ligation-dependant probe amplification (RT-MLPA). Our data and previously published data [5,10,11,12] were compiled in the Table S3. 

**Figure 1:**
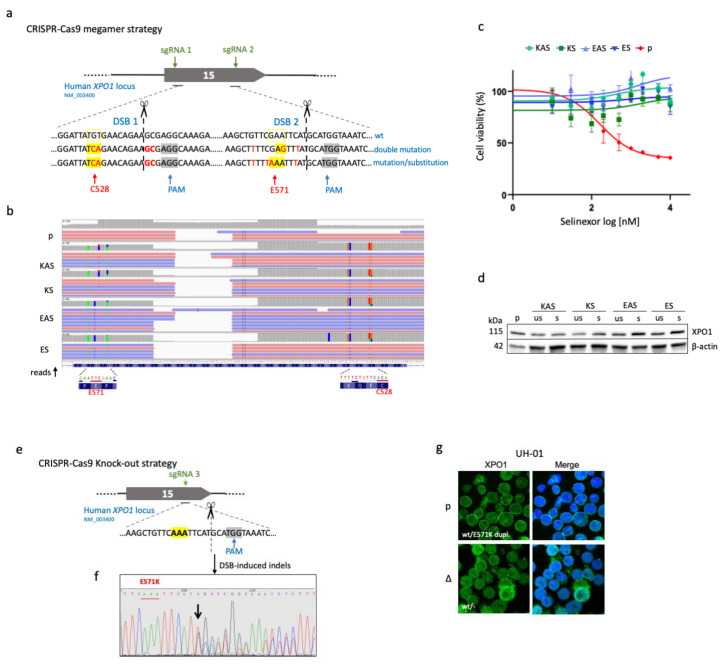
U2940-derived KAS and K clones expressed a wt and a C528S/E571K allele, UH-01Δ cells expressed only one wt allele. (**a**) For the CRISPR–Cas9 megamer strategy, we used two sgRNAs (in green) that respectively target the TGT and GAA codons (in yellow). We chose the PAMs (in grey) closest to the codons C528 and E571 to ensure a high HDR efficiency. After sgRNA 1, sgRNA 2 and PAM recognition, two double-strand breaks (DSBs) were generated by the Cas9 and cells undergoing HDR for megamer insertion between the DSBs. We used a 400 bp megamer that contained the C528S and E571K mutations and silent mutations serving as barcodes and preventing a second recognition of the modified target sites by the sgRNA (Tables S4 and S5). As a control we used a megamer containing the C528S mutation and the E571E substitution and silent mutations (Table S5). (**b**) The clones KAS (antisense orientation)/KS (sense orientation) and EAS (antisense orientation)/ES (sense orientation) generated by the CRISPR–Cas9 technique and the parental (p) cell line were analysed by NGS. The results are illustrated with an IGV (Integrative Genomics Viewer) view. The E571K and C528S XPO1 mutations (in red) are pointed out along the sequence with the silent mutations (in black) introduced. The numbers of reads are on the y-axis. The nucleotides are indicated on the x-axis. The sequence is reversed 3’–5’. The proportion of each nucleotide is represented as a coloured bar (A, in green; C, in blue; T, in red; G, in orange). (**c**) Two weeks after the transfection, the four batches of KAS/KS and EAS/ES cells were cultured in the presence of selinexor (1 µM) for selection. One week later, cells were assessed for selinexor sensitivity/resistance with an MTS test. Selinexor-selected cells and U2940 parental cells (in red) were treated for 48 h with various concentrations of selinexor (0.01–20 µM) and cell viability was calculated. The experiment was performed once (due to a limited number of cells); each culture condition was done in triplicate. (**d**) XPO1 expression was analysed in selected (s) and unselected (us) cell batches along with the parental (p) U2940 cell line by western blotting (WB) with the indicated antibodies (Abs) (Table S7). An anti-β-actin Ab served as a control of charge and transfer. The anti-XPO1 Ab is directed against the C-terminal part of the XPO1 protein (residues 772–1071). (**e**) Schematic representation of the CRISPR–Cas9 knockout (KO) strategy set up for UH-01 cHL cells. We used the sgRNA 3 (Table S4, green arrow) that targets the E571K codon (in yellow) to delete the mutated E571K allele. The PAM site (in grey) was the same as the one chosen previously in (a). The E571K codon is highlighted in bold. (**f**) Three days after nucleofection, gDNA was sequenced by the Sanger method. The genomic sequence was modified by the insertion of nucleotides (arrowed) three bases upstream the PAM sequence allowing the generation of indels. (**g**) The expression of XPO1 was analysed by immunofluorescence (IF) in edited (Δ) and parental (p) cells with an anti-XPO1 Ab directed against the C-terminal part of the protein (residues 1025–1071) (Table S7). In turn, only the XPO1 protein encoded by the wt allele could be detected. Cells were counterstained with 4′,6-diamidino-2-phenylindole (DAPI, in blue) and analysed by confocal microscopy (×180 magnification).

We used the CRISPR–Cas9 technology to establish several edited PMBL and cHL cells (see Supplementary Protocol and Supplementary Results). We first optimised a CRISPR-barcoding technique that allowed us to introduce the E571K mutation in the U2940 cell line (*XPO1*^wt^) and to follow the population of edited cells (Figure S1a–c). However, despite the fact that the percentage of cells experiencing homology-directed repair (HDR) was correct (≈42%, Figure S1d), the number of edited cells decreased during the time-course of culture (Figure S1e). To select and enrich U2940 cells having integrated the *XPO1*^E571K^ mutation, we took advantage of the fact that *XPO1*^C528S^-mutant T cells are resistant to selinexor, an inhibitor of XPO1 [13]. We designed a strategy for introducing in the same cell the two close C528S and E571K mutations using megamers and two sgRNAs (Figure 1a, Table S4). 

We designed single-strand oligonucleotides (ssODNs) bearing both C528S and E571K mutations (or C528S mutation and E571E substitution as a control) and silent mutations serving as barcodes and sense/antisense orientations for each construct (Table S5). Cells were transfected by nucleofection, and the resulting cells are referred to as KS and KAS and for the sense and antisense double mutants, respectively; ES and EAS for the corresponding controls. Cells were treated with selinexor. Two weeks later, the presence of mutations was verified by next-generation sequencing (NGS, Figure 1b). A sub-population of cells resistant to selinexor contained the two C528S/E571K mutations or the C528S/E571E mutation/substitution (Figure 1b,c) and expressed the XPO1 protein (Figure 1d). Edited KAS and KS cells were heterozygous for the C528S and E571K mutations, as determined by pyrosequencing, whereas as EAS and ES cells were homozygous for the C528S mutation (Table S6). We further used another CRISPR–Cas9 strategy to delete the mutant E571K allele in UH-01 cHL cells (Figure 1e). Insertions/deletions (indels) of the targeted region of the *XPO1* gene were confirmed by Sanger sequencing (Figure 1f). As estimated by the Surveyor assay, at least 28% of *XPO1* alleles experienced non-homologous end-joining (NHEJ, Figure S1f). The wild-type form of XPO1 was synthesised in UH-01-edited cells (referred to UH-01Δ, Figure 1g). The *XPO1* gene was reported to be necessary for cell survival [14,15]. UH-01Δ cells expressing only one wt *XPO1* allele were viable, although they grew slowly compared to the parental cells.

### 2.2. XPO1^E571K^ Mutation is Present at the mRNA and Protein Levels

**Figure 2:**
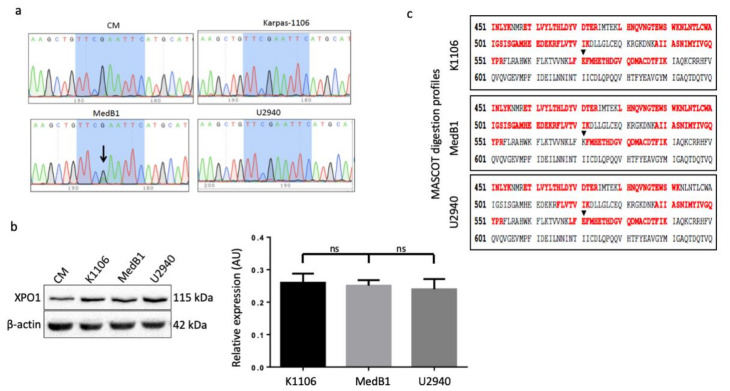
XPO1^E571K^ mutation is present in the mRNA and protein of MedB1 cells. (**a**) Total RNAs were purified from PMBL and CM cells. The relevant region of the XPO1 gene was amplified by RT-PCR with the primers presented in the Table S8. XPO1-PCR fragments were sequenced using the Sanger method. The resulting profiles are shown. The mutation present in MedB1 cells is arrowed. (**b**) Whole-cell proteins were purified from cultured cells, separated on SDS-PAGE, transferred onto nitrocellulose sheets. Blots were cut in strips and incubated with an anti-XPO1 Ab. An anti-β-actin Ab was used as a control of loading and transfer (Table S7). The experiment was done three times and the level of XPO1 protein expression was estimated by densitometry. ns, not significant with the *t*-test. (**c**) Whole-cell protein extracts were obtained from the three PMBL cell lines. XPO1 was immunoprecipitated with an anti-XPO1 Ab (70% of total proteins were immunoprecipitated using 0.15 μg of Ab as evaluated in Figure S2b), processed and submitted to mass spectrometry (MS). MS-sequenced peptides from XPO1 found by Mascot database matches are shown in red over the theoretical sequence in black. Black triangles indicate position 571 of the three sequences from the three cell lines. The Mascot minimum scored over 25 when the minimum Mascot score was 16 for *p* < 0.05. Relevant mass spectra annotations are available in Figures S3–S5.

To test whether the mutant gene is expressed in MedB1 cells (XPO1^wt/E571K^), we used RT-PCR and amplified the relevant region in PMBL cells with the CM untransformed B-cell line as a control (Figure S2a). XPO1-PCR amplified fragments were next sequenced by the Sanger method. The nucleotide G (arrowed) was replaced by both an G and an A only in MedB1 cells (Figure 2a). This change corresponded to the chr2:g61719472C>T mutation described previously [4]. 

Whole-cell proteins were purified from PMBL and CM cells and analysed by WB. The level of XPO1 protein was similar in the PMBL cells and higher than in the control non tumoral CM cells (Figure 2b). We next optimized an immunoprecipitation (IP) protocol (Figure S2b) and XPO1 complexes were separated by SDS-PAGE. Subsequently, the most efficient IP conditions were used for trypsin digestion and mass spectrometry (MS) analysis on four biological replicates. Figure 2c shows tryptic digestion patterns for each cell line obtained out of the digested IP eluates after nano liquid chromatography–MS (see also Figures S3–S5). The black arrows point out position 571 for each covered cell line. A peptide spanning residues 568–589 showed up (as seen in the top and bottom panels) while a shorter peptide spanning residues 572–589 showed up (as seen in the middle panel) indicating that, indeed, the E571K mutation created a new tryptic cleavage site three amino acids farther than the one already present at the existing lysine 568 (N-term side of the new lysine 571). We thus concluded that the XPO1-mutated protein is expressed in MedB1 cells with the same detectable amount as the wt protein in the two other cell lines. 

### 2.3. Nuclear Export Function of XPO1 is Maintained in MedB1 Cells 

**Figure 3:**
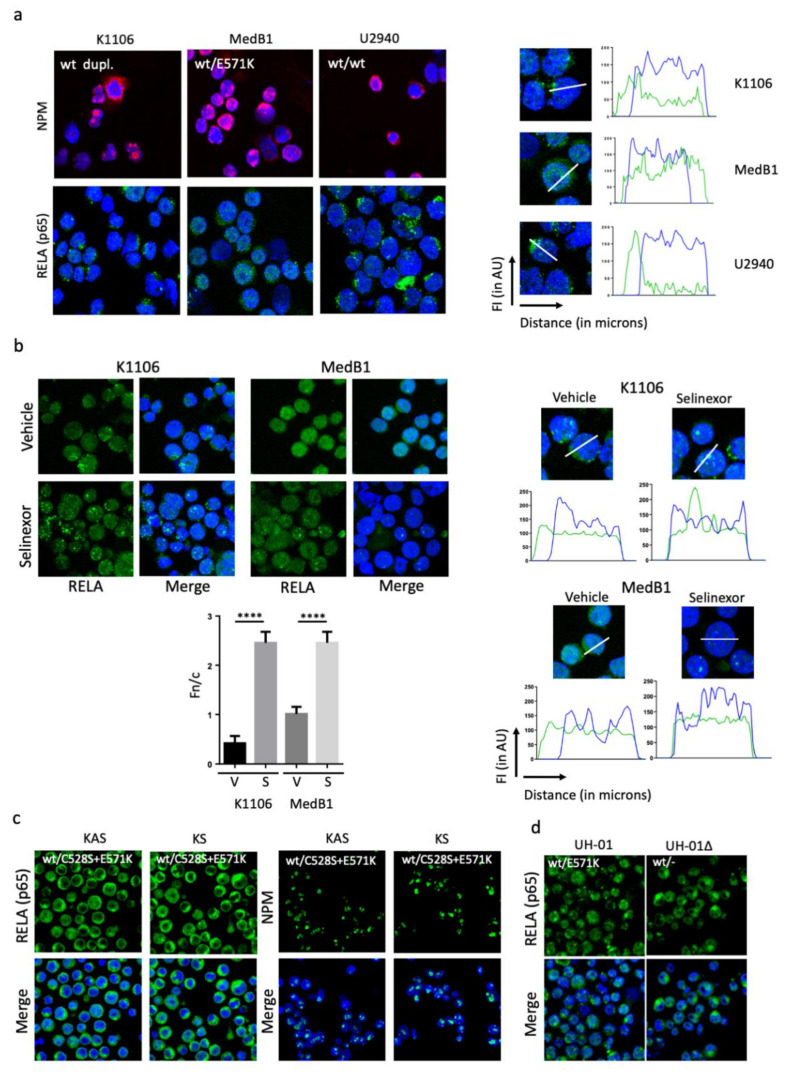
XPO1 is fully functional in PMBL cells. (**a**) PMBL cells were analysed by IF to determine the localisation of two XPO1 cargoes, NPM and RELA. We used primary Abs (Table S7) and a goat Alexa Fluor 633-conjugated anti-mouse IgG or a goat Alexa Fluor 488-conjugated anti-rabbit IgG as a secondary Ab. Slides were counterstained with DAPI and analysed by confocal microscopy (×180 magnification). For the RELA staining, images were processed with the ImageJ software and the fluorescence intensity of each fluorophore was plotted along a line crossing one representative cell (white line). Data were exported to generate the curves of fluorescence intensity (FI, in arbitrary units on the y-axis) as a function of the distance (left to right, in pixels on the x-axis). Regarding NPM staining, the experiment was done three times for MedB1 and U2940 cells and twice for K1106 cells. Regarding RELA staining, the experiment was done four times for the three cell lines. (**b**) K1106 and MedB1 cells were treated with 1 µM selinexor for 24 h then harvested. Cells were analysed by IF for RELA expression and localisation by confocal microscopy (×180 magnification) as described in (a). Images were processed with the ImageJ software and exported. The curves of FI as a function of the distance were drawn as previously described. The nuclear accumulation of RELA in selinexor-treated (S) vs. vehicle-treated (V) K1106 and MedB1 cells was quantified according to Kuusisto et al. [19], as described in Section 4.3. The Fn/c was calculated from six independent fields covering at least 100 cells. The experiment was done three times. Presented data are means ± SD. (**c**) U2940-derived KAS/KS clones were analysed by IF for RELA and NPM localisation as described in (a). (**d**) UH-01 parental and edited (Δ) cells were analysed by IF for RELA localisation as described in (a). Slides were analysed by confocal microscopy (×180 magnification). The experiments were done once due to the limited number of cells. ****, *p* < 0.001 with the *t*-test.

To verify whether the XPO1^E571K^ protein is fully functional, we analysed the localisations of two XPO1 cargoes known to be relevant for lymphoma pathology: nucleophosmin (NPM) and RELA [16,17]. Immunostaining and indirect IF revealed that NPM was strictly nuclear, whereas RELA was both nuclear and cytoplasmic whatever the XPO1 status (Figure 3a). Two main XPO1 partners are necessary for nuclear export, RanBP1 and RanBP2 [18]. Both are expressed in the three cell lines (Figure S2c) excluding a major default of the nuclear export apparatus. We next analysed the effects of selinexor on RELA localisation in K1106 and MedB1 cells. We observed a nuclear accumulation of RELA in selinexor-treated cells (Figure 3b) indicating that XPO1 was inhibited, and in turn, functional in both cell lines. The subcellular localisation of RELA and its nuclear accumulation in selinexor-treated K1106 and MedB1 cells was confirmed by image processing with the ImageJ software and the calculation of the Fn/c index. We further confirmed that RELA and NPM localisations were similar in U2940 (XPO1^wt^) and U2940-derived KAS and KS edited clones having a XPO1^wt/C526S/E571K^ gene (Figure 3c). Moreover, RELA distribution was similar in UH-01 parental and edited (Δ) cells (Figure 3d). Our data indicated that the localisation of XPO1 cargoes was not impacted by the insertion or the deletion of the mutant allele. The E571K mutation has no major effect on the nuclear export conducted by XPO1. 

### 2.4. Wild-Type and Mutant XPO1 Proteins Localised in Different Compartments in PMBL Cells

**Figure 4:**
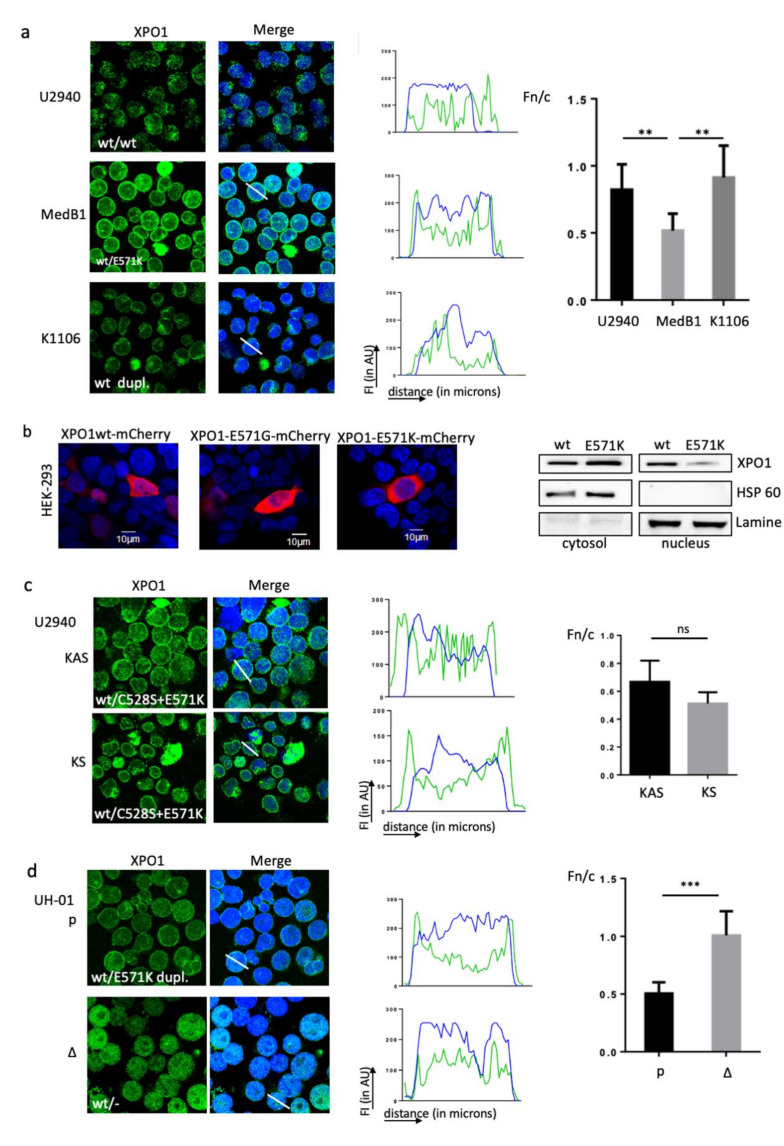
XPO1 accumulates at the perinuclear rim in XPO1^E571K^-expressing cells. (**a**) PMBL cells were analysed by IF to determine the localisation of XPO1. We used an anti-XPO1 Ab (Table S7) and a goat Alexa Fluor 488-conjugated anti-rabbit IgG as the secondary Ab. Slides were counterstained with DAPI and analysed by confocal microscopy (×180 magnification). Images were processed with the ImageJ software and data were exported to generate the curves of FI in AU as a function of the distance in pixels. The Fn/c was calculated from six independent fields covering at least 100 cells. The experiment was done five times. Presented data are means ± SD. (**b**) HEK-293 cells were transfected with plasmids expressing XPO1-mCherry fusion proteins. Cells were harvested 24 h after transfection and analysed for red fluorescence with confocal microscopy. The experiments were repeated four times. Cytosolic and nuclear extracts were prepared from HEK-293 cells transfected with the XPO1-mCherry-wt (wt) or XPO1-E571K-mCherry (E571K) plamids 48 h after transfection. Proteins were separated on SDS-PAGE and transferred onto nitrocellulose membranes. They were incubated with an anti-XPO1 Ab (Table S7). HSP60 and lamin A were used as specific markers for confirming the good cytoplasmic/nuclear separation and loading controls. (**c**) Selinexor-selected KAS and KS clones expressing the two C528S and E571K mutations were assessed for XPO1 localisation and images were processed as in (a). The experiment was done three times. Data are means ± SD. (**d**) Parental and UH-01Δ cells were assessed for XPO1 localisation and images were processed as described in (a) and the calculation of the Fn/c index. Due to the limited number of UH-01Δ cells, the experiment was done once. **, *p* < 0.01; ***, *p* < 0.001; ns, not significant.

We next analysed the subcellular distribution of XPO1 by IF. XPO1 was both nuclear and cytoplasmic in U2940 cells (XPO1^wt^) and K1106 cells (XPO1^dupl.^), whereas XPO1 accumulated at the nuclear membrane in MedB1 cells (XPO1^wt/E571K^) (Figure 4a). This was confirmed when images were processed with the ImageJ software and the calculation of the Fn/c index. To confirm that the E571K mutation modified XPO1 distribution, we transiently transfected the human HEK-293 cells with expression plasmids coding for wt, E571G (a mutation found in CLL [9]) and E571K XPO1-coupled to the mCherry fluorophore. XPO1^wt^ was both nuclear and cytoplasmic like the XPO1^E571G^ mutant, whereas XPO1^E571K^ mutant was mainly cytoplasmic (Figure 4b). The preferential cytosolic localisation of XPO1^E571K^ was confirmed with WB of cytoplasmic/nuclear differential protein extracts of transfected HEK-293 cells (Figure 4b). We further analysed the subcellular XPO1 localisation in U2940-derived KAS and KS clones in which one wt allele was replaced by a mutant E571K allele. In the clones, XPO1 tended to accumulate at the nuclear envelope (Figure 4c). Finally, we used parental (p) and edited (Δ) UH-01 cells. Compared to UH-01 parental cells, XPO1 was more diffuse and nuclear in UH-01Δ cells (Figure 4d). The processing of U2940 and UH-01 images with the ImageJ software and the calculation of the Fc/n index confirmed the XPO1 cytoplasmic accumulation in cells having the E571K mutant allele (MedB1, KAS/KS, UH-01, Figure 4a,c,d). These data indicated that the E571K mutation may alter XPO1 localisation. 

### 2.5. Mutant and Wild-Type XPO1 Possess Similar Interactomes

Using a proteomic approach, we analysed the XPO1^wt^ and XPO1^E571K^ interactomes in PMBL cells with the aim of observing either a different pattern of cargoes or specific cargoes. Following the previously described optimized IP protocol (Figure S2b), XPO1 complexes were separated and subsequently used for trypsin digestion and MS analysis on four biological replicates from the three PMBL cell lines. As a control, we set up four replicates in which immunoglobulins (Ig) of the same isotype replaced the anti-XPO1 Ab. The MS proteomics data (.Raw, .mgf and mascot .dat files have been deposited to the ProteomeXchange Consortium via the PRIDE [20] partner repository with the dataset identifier PXD016916 (http://www.proteomexchange.org/)) obtained with the U2940 cells were difficult to analyse because of an increased number of non-specific interactions, so we focused on the interactomes of K1106 and MedB1 cells that differ by the presence of one mutant allele (Figure S6a). By considering only the differentially expressed proteins (anti-XPO1 vs. Ig, *p* < 0.05), we found 20 specific XPO1-interacting proteins in K1106 cells, 13 in MedB1 cells, whereas 13 were common to both cell lines, including XPO1 (Figure S6b). We classified XPO1-interacting proteins using the STRING database tools (Figure S6c) and found the same GO terms: biological process (BP), molecular function (MF) and cellular component (CC), and the same INTERPRO and SMART protein domains, confirming that both interactomes are similar in the two cell lines. Despite the XPO1 duplication in K1106 and the XPO1 mutation in MedB1, the major role of XPO1 as a nuclear export protein is maintained (Table 1). Moreover, proteins previously described as XPO1 cargoes either by affinity capture or co-fractionation and MS [21,22,23] were sorted (Figure S6b). Other proteins not described as putative cargoes but belonging to a family of previously described cargoes (e.g., DDX41 vs. DDX1 or DDX5) were also characterized (Figure S6b). Thus, proteomics data validated our strategy and technical procedure.

### 2.6. XPO1^E571K^ Protein Binds the Karyopherin β1 at the Outer Nuclear Membrane

**Figure 5:**
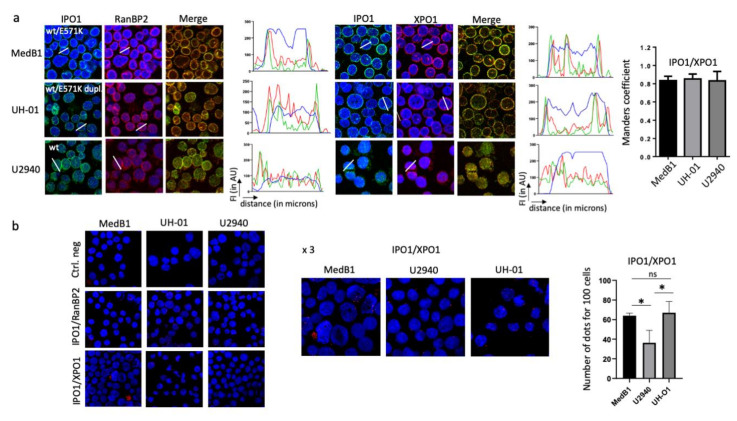

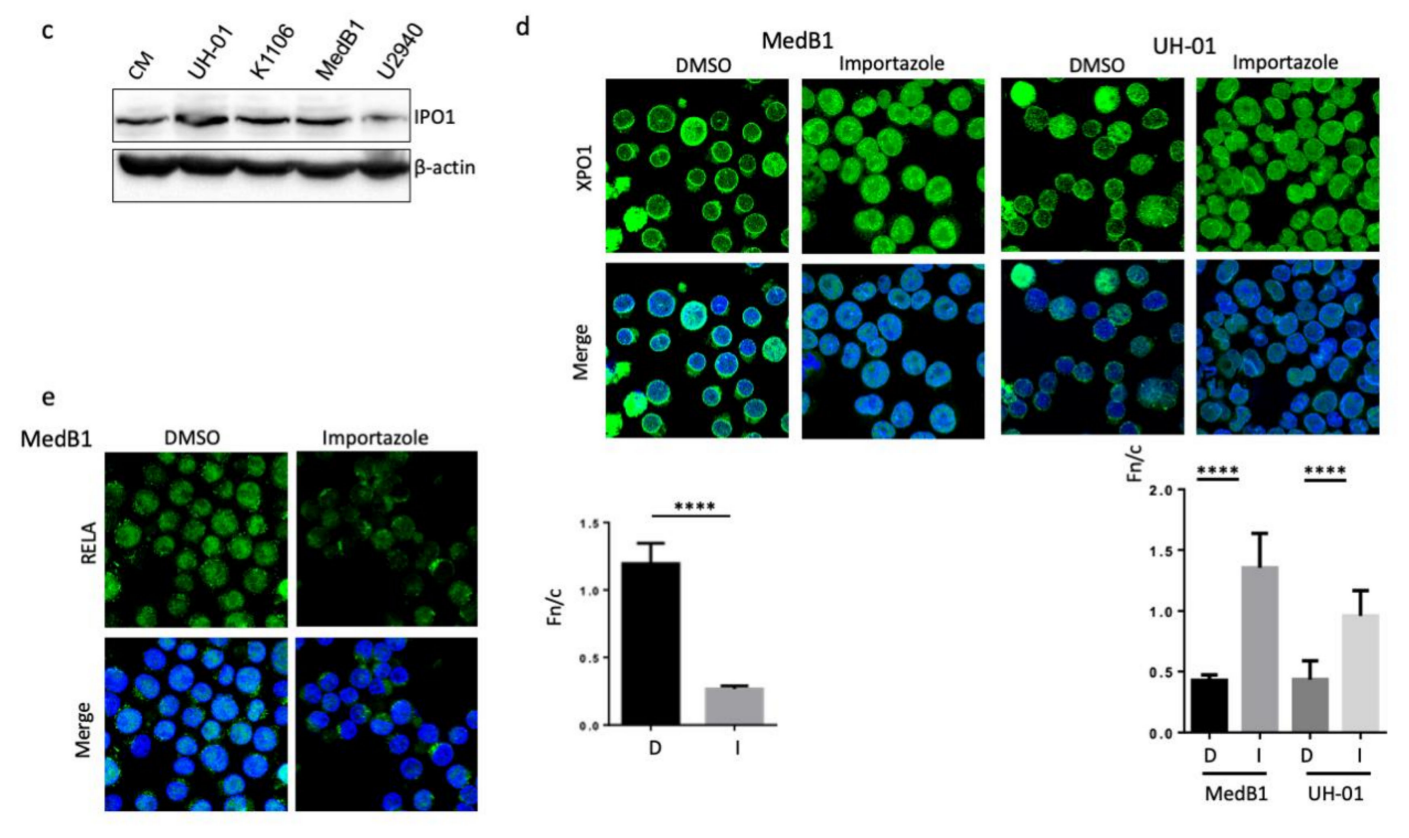
XPO1 and IPO1 are colocalised and bound in the PMBL and cHL cell lines and an importazole-treatment allows XPO1 to relocate to the nucleus. (**a**) MedB1, UH-01 and U2940 cells were analysed by IF to determine the colocalisation of XPO1 and RanBP2 (as a control) or IPO1. We used anti-XPO1, anti-RanBP2 and anti-IPO1 as primary Abs (Table S7) and goat Alexa Fluor 488- or 633-conjugated anti-rabbit IgG as secondary Ab. Slides were counterstained with DAPI and analysed with the ImageJ software as described previously. The colocalisation of XPO1 and IPO1 was confirmed with the Manders overlap coefficient from stained cells on three independent images for each staining condition (https://imagej.nih.gov/ij/). Means ± SD are indicated on the histograms. At least 30 cells per field and three fields per cell line were analysed. (**b**) The Duolink PLA technology was used on the same cell lines to investigate XPO1/IPO1 interactions with IPO1/RanBP2 interactions as a positive control. Slides were incubated with the primary Abs (Table S7), except for the negative control, and with the secondary Abs conjugated with the PLUS and MINUS probes. The ligation and amplification steps were next performed and the slides were counterstained with DAPI and observed with a confocal microscope (×180 magnification). Enlargements (×3) of positive slides are shown. Red dots were counted on at least 70 cells on each slide. For each experiment, two slides were set up, and three independent experiments ertr performed (Table S9). The means ± SD of red dot counts for 100 cells of all experiments are presented in the histogram. (**c**) Whole cell extracts were prepared from the indicated cell lines. Proteins were separated on SDS-PAGE and transferred onto nitrocellulose membranes. Membranes were then incubated with an anti-IPO1 Ab (Table S7). An anti-β-actin Ab was used for loading and transfer controls. (**d**,**e**) MedB1 and UH-01 cells were treated with vehicle (0.01% DMSO, D) or 4 µM importazole (I) for 24 h. Cells were then harvested and analysed by IF to determine the localisation of XPO1 (**d**) and RELA (**e**). We used an anti-XPO1 or an anti-RELA as the primary Ab (Table S7) and a goat Alexa Fluor 488-conjugated anti-rabbit IgG as the secondary Ab. Slides were counterstained with DAPI (merge) and analysed by confocal microscopy (×180 magnification). The Fn/c was calculated from six independent fields covering at least 100 cells for importazole (I) or vehicle (D)-treated MedB1 and UH-01 cells. The experiment was done twice. Presented data are means ± SD. ****, *p* < 0.0001 with the *t*-test.

Two studies reported previously that the *XPO1* mutation alters NES recognition and favours the export of cargoes with negatively charged C-terminal NES sequences [24,25]. Moreover, Taylor and co-workers described an enrichment of cytoplasmic vs. nuclear proteins in pre-B NALM6 cells bearing a mutant E571K allele [25]. We compared our data and those previously reported and found nine proteins common to NALM6^wt/E571K^ and MedB1 (Figure S7) Although these proteins are potentially interesting and relevant for PMBL and cHL pathologies (Supplementary results, Section 2.2), we focused on karyopherin β1 (KPNB1), also known as importin 1 (or IPO1). The import of proteins containing a nuclear localisation signal (NLS) requires a heterodimer of importin α and β subunits. Importin α binds the cargoes in the cytoplasm and IPO1 docks the complexes at the cytoplasmic side of the nuclear pore complex (NPC). In the presence RanGTP, the complex moves into the NPC and the importin subunits dissociate. RanBP2 (or NUP358) is the major nucleoporin component of the cytoplasmic filaments of the NPC and interacts with the FG-rich domains of IPO1 [26]. We used this property to analyse by IF the colocalisation of IPO1 and RanBP2. As observed in Figure 5a, in all analysed cells, the red fluorescent signal (RanBP2) and the green fluorescent signal (IPO1) peaked together, and the intensity of the signals is high outside the nucleus confirming the cytoplasmic localisation of IPO1. Analysing XPO1 and IPO1, we found that both proteins colocalised at the cytoplasmic face of the nuclear envelope. The calculated Manders’ overlap coefficients were 0.834 ± 0.049, 0.830 ± 0.053 and 0.850 ± 0.088 for MedB1, UH-01 and U2940 cells, respectively, confirming the colocalisation of XPO1 and IPO1 (Figure 5a). 

The direct binding of IPO1 and XPO1 was further confirmed by the proximity-ligation assay (PLA). In the negative control, no red dot was detected, whereas in the positive control red dots were observed (Figure 5b). Our results are in good agreement with the known respective roles of IPO1 and RanBP2 and their interactions [27]. Red dot blots, each one corresponding to one XPO1/IPO1 interaction, were also observed in the three PMBL/cHL cell lines tested, independently of *XPO1* status (Figure 5b). However, according to IF data, dots were mostly nuclear in U2940 cells and cytoplasmic in MedB1 and UH-01 cells. We counted the number of dots for 100 observed cells from the three cell lines. We found that the number of dots (i.e., XPO1/IPO1 interactions) was statistically higher in the two cell lines having the E571K mutation compared to U2940 cells having only wild-type *XPO1* alleles (Figure 5b). 

With the levels of XPO1 and IPO1 being similar in all cell lines (Figure 5c), we hypothesised that the increased number of dots between the two proteins could be due to stronger interactions between the two partners. We finally treated PMBL/cHL cells with importazole, a small molecule inhibitor of the nuclear transport receptor that blocks IPO1-mediated nuclear import without disrupting XPO1-mediated nuclear export [28]. We then analysed by IF the sub-cellular localisation of XPO1 in cytoplasmic/nuclear extracts obtained from MedB1 and UH-01-treated cells and -untreated cells (Figure 5d). Importazole-treatment dissociates the XPO1/IPO1 complexes, allowing XPO1 to relocate into the nucleus. These data confirmed that mutant XPO1 localisation is dependent on IPO1. Finally, we analysed RELA, known to possess a classical nuclear localisation signal (NLS) and to be a cargo of KPNA/IPO1 [29,30]. Due to the constitutive activation of the NF-κB signalling pathway in MedB1 cells, a common feature of PMBL [31], RELA localised into the nucleus in MedB1 cells (Figure 5e). Upon importazole-treatment, RELA accumulated in the cytoplasm confirming the inhibition of the nuclear import and its functionality. 

## 3. Discussion

The present work focused on the understanding of the functional impact of the “hot-spot” *XPO1*^E571K^ mutation in B-cell lymphoma. Although *XPO1* amplification was observed in both solid and haematological malignancies, the *XPO1*^E571K^ mutation seems lineage-specific and arises almost exclusively in B cells [4,5,9,25]. We first verified that the E571K mutation was present in the XPO1 protein. We next found that the mutant XPO1 protein displayed an altered cellular localisation in PMBL/cHL cell lines and their CRISPR–Cas9-edited counterparts. Indeed, using several CRISPR–Cas9 strategies, we introduced the E571K mutation in the U2940 cell line (*XPO1*^wt^) and deleted the mutant allele in the UH-01 cell line (*XPO1*^wt/E571K^). In KAS and KS cells derived from U2940 cells, XPO1 that shuttled between the nucleus and the cytoplasm in the parental cells accumulated at the nucleocytoplasmic membrane, whereas in UH-01Δ cells with no mutant allele, XPO1 was freed from the nuclear rim and became nuclear. The analysis of XPO1 interactome with a proteomic approach of cells having or not the XPO1 mutation revealed a network of similar proteins, confirming that the nuclear export was not intensively modified, in agreement with in vitro and in vivo reported data [24,25]. However, we found some proteins uniquely present in *XPO1*^E571K^-bearing cells. The preferential binding of mutant XPO1 with the importin β1 or IPO1 reported here may explain its abnormal localisation. 

The nuclear export and import of cargoes through the nuclear pore complex are mediated by carrier proteins known as export and import receptors of the karyopherin-β family [32,33]. Importins carry a wide range of cargoes, including those having classical nuclear localisation signals (NLSs), into the nucleus, whereas exportins carry cargoes containing nuclear export signals (NESs) to the cytoplasm. This receptor-mediated transport is orchestrated by RanGTP, which dissociates cargoes from importins, but conversely is required for cargo binding to exportins [32,33]. Within the nucleus, XPO1 recognises the nuclear export signal of cargoes; this interaction is then stabilised by RanGTP. Due to a Ran-GTP/Ran-GDP gradient dependent on the localisation of RCC1 (regulator of chromosome condensation 1, the Ran guanine exchange factor) and RanGAP (the Ran activating protein) in the nucleus and in the cytoplasm, respectively, XPO1-cargo-RanGTP complexes go through the NPC. This process is facilitated by their binding to nucleoporins (Nups), including Nup98 at the nucleoplasmic side and Nup214-Nup88 at the cytoplasmic side [34]. In the cytoplasm, XPO1-cargo-RanGTP complexes encounter RanBP1 and RanBP2 (or Nup358) which facilitate the hydrolysis of RanGTP and the cargo release [34]. The accumulation of mutant XPO1 at the nucleoplasmic side could be due to a default of cargo release imposed by a stronger affinity of XPO1 for one (or more) protein of the export complex when it dissociates. Human XPO1 is a ring-shaped protein composed of 21 HEAT repeats, formed by two helixes α and β, separated by a loop. The hydrophobic pocket of XPO1 at the surface of the protein, recognising the NES, is formed by H11 and H12 [35,36]. The H1-6 HEAT repeats and a loop on H9 of XPO1 contribute to the binding of RanGTP [37]. Therefore, the E571K mutation present on H12 cannot modify directly the affinity of XPO1 for RanGTP, but could do so allosterically. We did not find any mislocalisation of RanBP1 in PMBL cells including MedB1 (Figure S2c). Finally, using the available crystal structures of XPO1 and IPO1, each in complex with partner proteins, we attempted to construct models of ternary complexes (Figure S8). The model, obtained by superimposition of RanBP2 from the two structures, shows that XPO1 and IPO1 use the same interface, which rules out the hypothesis that the interaction can occur in this manner (Figure S8). The last possibility is a direct binding between XPO1 and IPO1 that could be altered by the presence of the E571K mutation.

The description of XPO1 recurrent mutations in a large percentage of PMBL and cHL patients suggests that XPO1^E571K^ conveys oncogenic functions. This is also highly sustained by the observation that mutations persist in patients without any selection pressure. Taylor and colleagues reported that the E571K mutant form of XPO1 is a driver of B-cell transformation [25]. When expressed in a pre-B cell line, the heterozygous mutation induces in vivo and in vitro the proliferation of transfected cells. By contrast, XPO1^E571K^ inhibits HEK-293 cell growth and causes mitotic defects [38]. When analysing the proliferation properties of KS an KAS clones in which we introduced the double C528S and E571K mutations, we found no differences compared with the parental U2940 cells (Figure S9). A direct role of XPO1^E571K^ in the control of proliferation is still an open question. Alternatively, the oncogenic properties of the mutant XPO1^E571K^ protein could rely on alterations of cargoes binding. Although the E571K mutation could increase the XPO1 affinity for NES sequences bearing negatively charged residues, as reported in vitro [24], there is no experimental evidence that XPO1^wt^ and XPO1^E571K^ interactomes are different ([25] and our present data). Moreover, most NESs similarly bind XPO1^wt^ and XPO1^E571K^ proteins, and the presence of charged residues within the NES cannot predict any differences for binding [38]. Our data suggest another mechanism. The binding of XPO1^E57K^ to IPO1 modifies its subcellular localisation, and in turn, the shuttling of some cargoes, potentially including oncoproteins and tumour suppressors. Further studies are necessary to investigate the dynamics of export and import complexes in relation to the E571K mutation.

There is a growing interest in targeting XPO1 in cancer treatment, and various SINEs have been developed, including Selinexor, and are still under development [7,39]. Selinexor exhibits a great therapeutic efficacy either alone or in combination therapies and is currently being assayed in numerous clinical trials (//clinicaltrials.gov/). However, IPO1 could also be considered a relevant therapeutic target [40]. IPO1 also appears important for B-cell and plasma cell diseases. Indeed, upregulation of the protein contributes to promote cell proliferation and tumour microenvironment-mediated resistance [41]. IPO1 mediates NF-κB transduction in the nucleus of MM cells and controls proliferation and apoptosis [42]. XPO1 inhibition blocks the nuclear export and allows tumour suppressors proteins to relocate into the nucleus and to recover their cell functions. We report here that IPO1 inhibition allowed the nuclear relocation of XPO1 for cells expressing the mutant protein, opening up new therapeutic perspectives. 

## 4. Materials and Methods 

### 4.1. Cell Culture and Transfection

The PMBL cell lines K1106, U2940 and MedB1 and the cHL UH-01 cell line have been described previously [4,5]. UH-01 (ACC-626) and U2940 (ACC-634) cells were purchased from DSMZ (Leibniz, Germany). MedB1 and K1106 cell lines, a generous gift of Karen Leroy, were authenticated by STR profiling (DSMZ). The untransformed CM cell line used as a control is a mature B-cell line immortalized by the Epstein–Barr virus. 

Cell lines were cultured in RPMI 1640 or IMDM medium (Lonza, Basel, Switzerland) supplemented with 10–20% foetal calf serum (FCS, PAA laboratories, Pasching, Austria), 2 mM L-glutamine and antibiotics (Lonza), under a humid atmosphere at 37 °C. Cells were regularly checked for mycoplasma contamination; moreover, each batch of cells was maintained in culture less than three months. The human HEK-293 cell line was maintained in DMEM medium (Lonza) supplemented with 10% FCS, L-glutamine and antibiotics. 

U2940 and UH-01 cells transfections were performed by nucleofection (4D-Nucleofector, Lonza, Basel, Switzerland). We used the Cell Line Optimisation 4D-Nucleofector X kit and the pmaxGFP vector as positive control. The NucleoCounter NC-3000 (ChemoMetec, Allerød, Denmark) was used to determine viability and transfection efficiency. We selected the best conditions regarding viability and transfection efficiency and used the SF solution and the DS-150 and DN-100 programs for U2940 and UH-01 cells, respectively. HEK-293 cells were transfected by the phosphate calcium method, as described in [43]. 

### 4.2. Cell Viability Assay

Cells were seeded in 96-well plates in complete medium (2 × 10^5^ cells per well) and treated for 48 h with vehicle (0.01% dimethylsulfoxide, DMSO) or various concentrations of selinexor (KPT-330, 0.01–20 µM, Selleckchem, Houston, TX, USA). Cell viability was quantified using an MTS assay (CellTiter 96 AQ_ueous_ One Solution Cell Proliferation Assay, Promega, Madison, WI, USA) according to the manufacturer’s instructions and as previously described [44]. The index of cytotoxicity (IC_50_) was calculated with the Prism v8.0 software (GraphPad, San Diego, CA, USA). 

### 4.3. Indirect Immunofluorescence and Confocal Microscopy

Cells were cytospun on superfrost glass slides, fixed in 4% paraformaldehyde, and permeabilized in 0.5% Triton-X100. The slides were then stained with primary antibodies (Abs), and with Alexa Fluor 488- (in green) or 633- (in red) conjugated goat anti-mouse or anti-rabbit IgG as secondary Abs (Invitrogen, Carlsbad, CA, USA), and counterstained with DAPI (in blue, Molecular Probes, Eugene, OR, USA). The primary Abs used in this study are presented in Table S7. The slides were observed with a confocal microscope (Fluoview FV100, Olympus, Rungis, France). The fluorescence intensity (in arbitrary units, AU) of each fluorophore was estimated with the ImageJ software (available from https://imagej.nih.gov/il/) as described previously [44]. Quantification of nuclear and cytoplasmic distribution of cargo proteins was performed as described by Kuusisto et al. [19]. Briefly, using ImageJ software, three mean fluorescence intensities—Fc for cytoplasmic fluorescence, Fn for nuclear fluorescence and Fb for background fluorescence—were determined by drawing a region of interest (ROI) of 30 arbitrary units in each compartment of each analysed cell. The ratio of nuclear to cytoplasmic fluorescence Fn/c was determined according to the following formula: Fn/c = (Fn - Fb) / (Fc - Fb). The mean fluorescence intensity (MFI) of each experimental condition was used to draw the histograms with the Prism software (v6.0, GraphPad). 

### 4.4. Proximity Ligation Assay

The proximity ligation (PLA) assay was used to confirm protein interactions. We used the Duolink In Situ Red Starter Kit (DUO92101, Sigma-Aldrich, Saint-Louis, MO, USA), according to [44], using primary Abs (Table S7) and as secondary Abs the PLUS and MINUS probes. As a positive control we used an anti-RanBP2 Ab, RanBP2 being a well-known partner of XPO1 [18]. As a negative control, no primary Ab was added in the reaction mixture. The slides were observed with a confocal microscope (Fluoview FV 100, Olympus). 

### 4.5. Western Blotting 

Whole-cell protein extracts were prepared from exponentially growing cells. Cells were lysed with a lysis buffer containing 1% NP40, 10% glycerol, 0.05 M Tris pH7.5, 0.15 M NaCl and a cocktail of protease and phosphatase inhibitors. Insoluble material was discarded and soluble proteins were recovered and quantified. Nuclear and cytoplasmic extracts were prepared with the NE-PER Nuclear and Cytoplasmic Extraction reagent kit (Thermo Fisher Scientific, Waltham, MA, USA) according to the manufacturer’s instructions. The methods used for SDS-PAGE and WB have been described previously [45]. The antibodies used are described in Table S7. Levels of detected protein were quantified using the ChemiDoc XR+ and the ImageLab software (Bio-Rad, Hercules, CA, USA). 

### 4.6. Immunoprecipitation, Mass Spectrometry and Protein Characterisation

The optimisation of IP conditions is described in Figure S2b. Briefly, U2940 cells were lysed in a buffer containing 25 mM Tris-HCl pH7.5, 150 mM NaCl, 5 mM EDTA, 10% glycerol, 0.5 % NP40 and a cocktail of protease and phosphatase inhibitors (250 μL for 10^7^ cells). IP of XPO1 was performed with 15 µL of Dynabeads Protein A and 0.15 µg of anti-XPO1 Ab (PLA0109, Sigma-Aldrich) per mg of total protein extract. XPO1 was eluted in a buffer containing 50 mM Tris (pH8.5), 2% SDS, 20 mM Tris(2-carboxyethyl)phosphine and 50 mM chloroacetamide, heated for 5 min at 95 °C and trypsin-digested overnight using S-trap micro spin columns according to the manufacturer’s instructions(www.protifi.com). Eluted peptides were dried in a vacuum centrifuge (SpeedVac, Eppendorf, Montesson, France) and solubilised with 10 µL of a 10% acetonitrile (ACN), 0.1% trifluoroacetic acid (TFA) in milliQ-H_2_O solution. 

Nano-liquid chromatography and MS analyses were performed on a 2 h run per replicate as previously described [46]. The mass spectrometry experimental data were compared with theoretical data using Mascot v2.5.1 (Matrix science) on a Homo sapiens (23,076 sequences) SwissProt protein database (October 2018) and an in-house database containing the E571K sequence variant of XPO1. The enzyme specificity type was trypsin’s (C-term side of K or R), and up to 1 missed cleavage was permitted. The precursor mass tolerance was set to 4 ppm and the fragment mass tolerance to 20 mmu. Carbamidomethylation of cysteins was set as constant modification and oxidation of methionines was set as variable modification. The best mass spectra annotations of three relevant peptides are available as Figures S3–S5.

### 4.7. CRISPR–Cas9 Editing

Depending of our objective: insertion of the E571K mutation (or the corresponding E571E as a control) in U2940 cells, insertion of the two C528S/E571K mutations in U2940 cells, or the KO of E571K in UH-01 cells, we set up various CRISPR–Cas9 strategies (Figure S1a, Figure 1a–e). sgRNA target sequences (Table S3) were designed using the CRISPR design tool hosted by the MIT (//crispr.mit.edu) to minimize potential off-target effects. The sequences of the ssODNs (Integrated DNA Technologies or IDT, Coralville, IA) containing functional and silent mutations used as genetic barcodes (Table S4) were chosen according to Guernet et al. [47]. Two types of ssODNs were used: either ultramers (DNA sequences of less than 200 bp) or megamers (DNA sequences of about 400 bp). For editing lymphoma cells, CRISPR–Cas9 experiments were performed with the Alt-R CRISPR–Cas9 system based on the use of a recombinant Cas9 (Alt-R S.p. Cas9 Nuclease 3NLS, #1074181, IDT). Transfections were performed with 0.4 μg of ribonucleoprotein (RNP) and 50 µM ssODN templates (Table S4) assembled in vitro as recommended by the manufacturer. Briefly, 200 µM sgRNAs (IDT) were incubated at 95 °C for 10 min. The RNP complex was formed by mixing sgRNAs with Alt-R™ S.p Cas9 Nuclease 3NLS at a final concentration of 5 µM. One microliter of 100 µM Alt-R Cas9 Electroporation Enhancer (IDT) was added to ensure optimal delivery of the Cas9 complex. Transfections were performed using nucleofection. Cells were then cultured and amplified for analysis. Barcodes detection was then performed by qPCR using specific primers (Table S10). We also used the Surveyor Mutation Detection kit to detect *XPO1* mutations and/or deletions as recommended by the supplier (IDT), and the restriction fragment length polymorphism (RFLP) technique to assess HDR efficiency. We amplified by PCR the region containing the sequence 5’-GCT GTT CGA ATT CAT GCA TGG TAA AT-3’ containing one *Eco*RI restriction site (in bold) using specific primers (Table S8). The PCR products were subjected to *Eco*RI digestion and the fragments were run on agarose gel. Digested and undigested fragment were quantified using the ChemiDoc XR+ and ImageLab software (Bio-Rad, Hercules, CA). For *XPO*^E571K^ deletion, we used the same conditions; however, no megamer was added to the transfection mix with the aim of triggering only NHEJ. The control experiments and technical improvements are described in detail in the Supplementary Information.

### 4.8. DNA and RNA Extraction, RT-PCR and Sanger Sequencing

Genomic (g)DNA was purified from cultured PMBL cell lines with the NucleoSpin Tissue kit according to the supplier’s instructions (Macherey-Nagel, Düren, Germany). Total RNA was extracted from the same cultured cells using Trizol reagent (Invitrogen). The purity of nucleic acids was checked with the NanoDrop One (Thermo Scientific, Waltham, MA). 

cDNAs were generated from purified RNA using random primers and the GoScript reverse transcriptase according to the manufacturer’s instructions (Promega, Madison, WI). PCRs were then realised with the Platinium *Taq* DNA Polymerase kit (Invitrogen) and the primers described in Table S8. PCR fragments were gel-purified (Wizard SV Gel and PCR Clean-Up System, Promega) and directly sequenced by the Sanger method in both directions using the same primers. 

### 4.9. DNA and RNA Pyrosequencing

Reverse transcription was performed on 500 ng of total RNA using MMLV-reverse transcriptase according to manufacturer’ instructions (Invitrogen). PCR amplification was performed on 5 µL of cDNA or 100 ng of DNA, using 45 µL of a PCR mix containing 25 µL Red’y’Star Mix (Eurogentec, Liege, Belgium), 1 µL XPO1_F primer (RNA or DNA, 10 µM), 1 µL 5’-biotinylated XPO1_R_biot primer (10 µM) and 18 µL water, as follows: 6 min at 94 °C; 35 cycles (30 sec at 94 °C, 30 sec at 60 °C, 30 sec at 72 °C); 4 min at 72 °C; and cooling at 10 °C. Then, 20 µL of PCR products were analysed on a PyroMark Q24 platform (Qiagen, Venlo, The Netherlands) using sequencing primers with a dispensation order predefined with PyroMark Q24 software (v2.0.7) and following standard procedures. The sequences of primers are listed in Table S2.

### 4.10. Next-Generation Sequencing

NGS experiments were performed using Ion Torrent Personal Machine (PGM) and variant analysis was performed using an in-house generated bioinformatics pipeline as previously described [48]. The gene panel used for the sequencing covers the exon 15 of *XPO1*, which contains the codons for E571 and C528 residues. 

### 4.11. Statistical Analyses

The Student’s *t*-test was used to determine the significance of differences between two experimental groups. Data were analysed in two-tailed tests, with *p* < 0.05 (*) considered to be significant.

## 5. Conclusions

XPO1 overexpression is commonly observed in solid cancers and haematological malignancies, leading to the aberrant localisations of tumour suppressors and cell cycle regulators, and to enhanced expressions of oncogenes associated with poor prognoses [7,39]. Interestingly, Ran, an XPO1/IPO1 partner, necessary for proper nucleocytoplasmic export and import, is also overexpressed and associated with a poor prognosis in solid cancers [49,50]. How the mislocalisation of XPO1 in B cells has a bearing on how the E571K mutation participates into the oncogenic process is still an open question. However, our data confirmed that aberrant nuclear export machinery could be regarded as a hallmark of oncogenesis.

## Figures and Tables

**Table 1:** Enrichment for XPO1 interactors identified in K1106 and MedB1 cells with STRING database.

K1106			
**GO Term**	**Biological Process**	**Count in Gene Set**	**False Discovery Rate**
GO:0046907	Intracellular protein transport	7/836	8.40 × 10^−4^
**GO Term**	**Molecular Function**	**Count in Gene Set**	**False Discovery Rate**
GO:0003924	GTPase activity	8/283	7.42 × 10^−7^
**GO Term**	**Cellular Component**	**Count in Gene Set**	**False Discovery Rate**
GO:0031090	Organelle membrane	20/2828	1.66 × 10^−7^
	**INTERPRO Prot. domains**	**Count in Gene Set**	**False Discovery Rate**
IPR001806	Small GTPase superfamily	7/171	6.80 × 10^−7^
	**SMART Prot. domains**	**Count in Gene Set**	**False Discovery Rate**
SM00175	Rab subfamily of GTPases	5/62	1.58 × 10^−6^
**MedB1**			
**GO Term**	**Biological Process**	**Count in Gene Set**	**False Discovery Rate**
GO:0046907	Intracellular protein transport	10/1390	2.20 × 10^−3^
**GO Term**	**Molecular Function**	**Count in Gene Set**	**False Discovery Rate**
GO:0003924	GTPase activity	4/283	3.60 × 10^−7^
**GO Term**	**Cellular Component**	**Count in Gene Set**	**False Discovery Rate**
GO:0031090	Organelle membrane	16/2828	2.84 × 10^−6^
	**INTERPRO Prot. domains**	**Count in Gene Set**	**False Discovery Rate**
IPR001806	Small GTPase superfamily	3/163	4.10 × 10^−3^
	**SMART Prot. domains**	**Count in Gene Set**	**False Discovery Rate**
SM00175	Rab subfamily of GTPases	3/62	1.10 × 10^−3^

The 33 proteins associated with XPO1 in K1106 cells and the 26 proteins associated with XPO1 in MedB1 cells, revealed by IP/MS, were analysed with the STRING v10.5 database (string-db.org/cgi/). Within the network of protein/protein interaction (PPI, Figure S6c), functional connections among the set of proteins were enriched and reported in the table with their GO pathway terms, the number of genes in their pathways and the false discovery rates calculated by Fisher’s exact tests followed by corrections of multiple testing.

## Supplementary Materials

The supplementary file is available online at https://www.mdpi.com/2072-6694/12/10/2829/s1. Figure S1: Optimisation of the CRISPR-barcoding strategy, Figure S2: Optimisation of the immunoprecipitation conditions and immunoflurescence images, Figures S3–S5: MS/MS annotated spectra from K1106, MedB1 and U2940 cells, respectively, Figure S6: Proteomic analysis of XPO1 interactome in PMBL cells, Figure S7: Comparison of XPO1 interactomes in K1106, MedB1 and NALM6 *XPO1*^wt/mut^, Figure S8: Hypothetical 3D-model of IPO1/XPO1/RanBP2 complex, Figure S9: Proliferation curves of U2940 cells, and KS/KAS and EAS/ES derivatives, Table S1: Relative quantification of mutant and wild-type (wt) *XPO1* alleles by pyrosequencing, Table S2: Sequences of the primers used for DNA and RNA pyrosequencing, Table S3: XPO1 status in the PMBL and cHL cell lines used in the study, Table S4: Sequences of sgRNA used in the CRISPR-Cas9 studies, Table S5: Sequences of ssODNs used in the CRISPR-Cas9 barcoding strategies, Table S6: Selinexor treatment allows the recovery of U2940 cells having the the two C528S/E571K mutations, Table S7: Primary antibodies used in the study, Table S8: Sequences of the primers used for RT-PCR and Sanger sequencing, Table S9: Proximity-ligation assay results, Table S10: Sequences of the primers used for PCR in CRISPR-Cas9 assays.
