# Supplementary material for: XPO1E571K Mutation Modifies Exportin 1 Localisation and Interactome in B-Cell Lymphoma
Source: Cancers (Basel). 2020 Sep 30;12(10):2829. doi: 10.3390/cancers12102829 (PMC7600770; doi:10.3390/cancers12102829)
Supplement: Supplementary file 1 [file cancers-12-02829-s001.pdf]

Supplementary Materials

XPO1^{E571K} Mutation Modifies Exportin 1 Localisation and Interactome in B-cell Lymphoma

Hadjer Miloudi, Élodie Bohers, François Guillonneau, Antoine Taly, Vincent Cabaud Gibouin, Pierre-Julien Viailly, Gaëtan Jego, Luca Grumolato, Fabrice Jardin and Brigitte Sola

1. Supplementary Protocol

1.1 PMBL and cHL Genome Editing Using Several CRISPR-Cas9 Strategies

Depending of our objective: insertion of the E571K mutation (or the corresponding E571E as a control) in U2940 cells, insertion of the two C528S/E571K mutations or the C528S/E571E mutation/substitution in U2940 cells, or the knock-out (KO) of E571K in UH-01 cells, we set up various CRISPR-Cas9 strategies. The sgRNA sequences (Table S4) as well as the ssODNs containing functional as well as silent mutations used as genetic barcodes (Table S5) were chosen according to Guernet et al. [1]. The pSpCas9(BB)-2A-Puro (PX459) plasmid (#48139, Addgene, Watertown, MA, USA) was used to express both Cas9 and the different sgRNAs, as previously described [2]. To check for strategy efficacy, HEK-293 cells were transfected with 2 µg of PX459 and 50 µM of either the *XPO1*^{E571E} or the *XPO1*^{E571K} ssODN templates (Table S5). Transfections were performed using calcium phosphate precipitation [3]; cells were then cultured and amplified before analysis. Barcodes detection was then performed by qPCR using primers specific for the wt or mutant alleles (Table S10). We used the Surveyor Mutation Detection kit to detect *XPO1* mutations and/or deletions as recommended by the supplier (IDT). We also used the restriction fragment length polymorphism (RFLP) technique to assess HDR efficiency. We amplified by PCR the region containing the sequence 5′GCT GTT C**GA ATT C**AT GCA TGG TAA AT-3′ containing one *Eco*RI restriction site (in bold) using specific primers (Table S8). The PCR products were subjected to *Eco*RI digestion and the fragments were run on agarose gel. Digested and undigested fragment were quantified using the ChemiDoc XR+ and ImageLab software (Bio-Rad, Hercules, CA, USA).

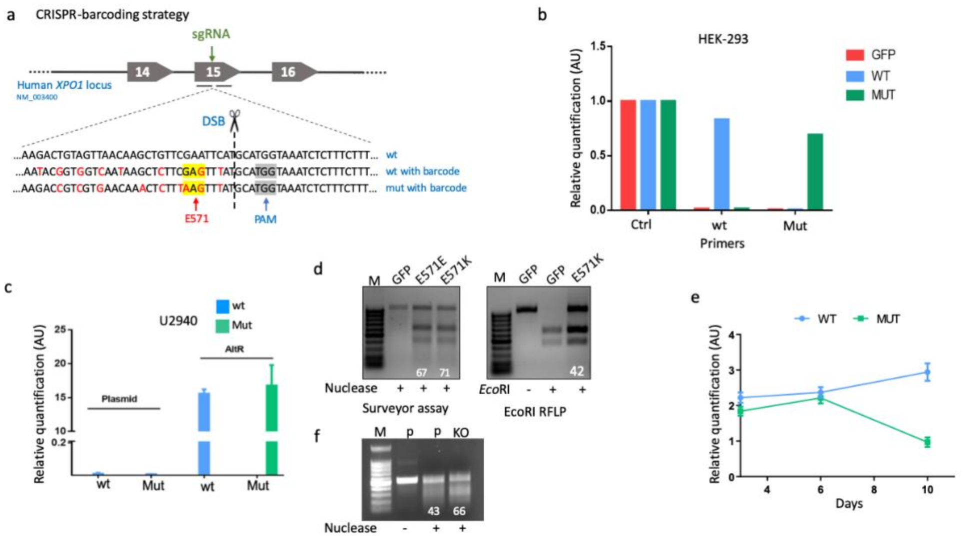

Figure S1. Optimisation of the CRISPR-barcoding strategy. (**a**) Schematic representation of the CRISPR-barcoding strategy. We used a single sgRNA that targets the E571 codon (green arrow) (Table S4) and an ultramer of 100 pb containing either the G > A mutation (mut) with a barcode or an A > G silent mutation (wt) with a barcode (Table S5). The PAM sites and the codon of interest (E571) are highlighted in grey and yellow, respectively. We chose the PAM site closest to the targeted GAA codon to increase the chances for HDR. After

recognition of the sgRNA and the PAM by the Cas9, a DSB is generated. In presence of single-stranded oligonucleotides (ssODN) containing barcodes and mutations (in red), HDR can occur allowing the insertion of nucleotides bearing one wt or mutant sequence and the associated barcodes. (**b**) HEK-293 cells were transfected by a Cas9-expressing plasmid specifically targeting *XPO1* to introduce either the wt (in blue) or mutant (mut, in green) sequences. We used GFP-transfected cells as a negative control (in red). gDNA was purified and qPCR was performed using three pairs of primers either outside of the targeted region (XPO1_ext) or amplifying the wt (XPO1_E571E) or the mutant sequences (XPO1_E571K) (Table S10) to detect specifically the barcode associated with either the wt or mutant ssODN. (**c**) U2940 cells were transfected either with the Cas9-expressing plasmid or the recombinant protein (AltR strategy) and gDNA was analysed by PCR using the same primers than in (b). (**d**) *XPO1* mutations/deletions were assessed by the Surveyor assay. E571K- or E571E-containing amplicons were annealed with an unmodified amplicon (200 ng of each) and digested with the Surveyor nuclease. The resulting products were run on an agarose gel and the intensity of each fragment measured by a ChemiDoc XRS+ imager and the ImageLab software. We estimated that, related to unmodified DNA, 67 and 71% of DNA was modified in *XPO1* E571K (Mut)- and E571E (wt)-bearing cells. No cleavage was detected in the GFP negative control. For the RFLP technique, we took advantage of the presence of an *Eco*RI restriction site in the genomic DNA within the *XPO1* region of interest. The insertion of a barcode in this region should destroy the restriction site in edited cells but not in non-edited cells. The region was amplified by PCR and fragments were subjected to *EcoR*I digestion. As shown in the GFP condition, all PCR fragments were digested, while in the E571K condition, some undigested PCR fragments were present. We quantified digested *vs.* undigested PCR fragments and evaluated HDR efficiency around 42%. (**e**) U2940 cells were transfected as in (c) and both populations (wt and mut) were independently followed with barcodes starting three days post-transfection for ten days. qPCR was performed on gDNA extracted from cultured cells at the indicated time points. The ratios of the mean value of the E571K barcode or E571E barcode signals relative to the XPO1 gene signal were plotted on the graph as a function of time (in days). (**f**) The surveyor assay was set up for parental (p) and UH-01Δ edited cells as described previously. As the UH-01 cell line is heterozygous for XPO1, heteroduplexes formed in the p condition, further considered as the control (100% of heteroduplexes). The heteroduplexes generated after theCRISPR-Cas9-induced knock-out of *XPO1* and the addition of nuclease were quantifying by densitometry and compared to the control.

2. Supplementary Results

2.1. The CRISPR Cas9 Strategy was Efficient for Introducing E571K Mutation in PMBL Cells

The CRISPR-barcoding technique allows to introduce a given mutation within the genome of cells and to trace a sub-population of genome-edited cells within a population of non-edited cells [1]. We used this approach to insert the single G > A mutation in the GAA codon encoding the glutamic acid 571 residue within the exon 15 of the *XPO1* gene, as well as additional silent point mutations serving as barcodes as schematised in Figure S1a. We validated the design of sgRNA and specific ultramers (Tables S4,S5) using the human HEK-293 cell line. Indeed, mutations were detected in the gDNA by PCR only in the corresponding transfection conditions (Figure S1b). Next, we transfected the U2940 cell line carrying two wt *XPO1* alleles. Cas9 was delivered using the PX459 plasmid as for HEK-293 cells, or as a recombinant protein (Alt-R system). The presence of the mutant (E571K)- and wt (E571E)-barcode sequences was analysed in the gDNA by PCR three days after transfection. The strategy using the Alt-R system proved to be remarkably more efficient compared to the plasmid (Figure S1c). We then estimated the CRISPR-Cas9 technique efficiency using the Surveyor assay and HDR using an RFLP technique. The Surveyor assay indicated a cleavage efficiency of 67% and 71% for E571E and E571K, respectively (Figure S1d). The percentage of HDR that occurred for ultramer insertion was estimated around 42% with an *Eco*RI RFLP assay (Figure S1d). We next used the barcodes for tracing edited cells bearing either the mutant E571K allele (in green) or the wt E571E allele (in blue) among the global cell population (Figure S1e). The proportion of edited cells was similar in both situations three days after the transfection and remained stable for three days more. Ten days after transfection, the fraction of cells bearing the E571K mutation although still present, showed a two-fold decrease compared to earlier time points. These results prompted us to develop another strategy for enriching the population of transfected cells with edited cells.

2.2. Proteomics Studies Reveal a Number of Membrane-Bound Proteins in XPO1^{E571K}-Expressing Cells

Apart from KPNB1/IPO1 that was studied in details, the other eight proteins that could preferentially interact with the mutant form of XPO1 were relevant and most of them are membrane-bound proteins. The *DHCR7* gene encodes the 7-dehydrocholesterol reductase enzyme that removes the C(7–8) double bond in the B ring of sterols and catalyzes the conversion of 7 dehydrocholesterol to cholesterol allowing the switch between cholesterol and vitamin D production. This gene is ubiquitously expressed and the protein localises to the endoplasmic reticulum (ER) membrane and nuclear outer membrane [4]. XPO1 and HSP90B1 binding was demonstrated by co-IP from HEK-293 cell lysates followed by nano-HPLC-MS/MS [5]. HSP90B1 (or glucose-regulated protein of 94 kDa, GRP94) is the ER-resident member of the heat-shock protein (HSP) 90 family and a major regulator of ER functions. As a molecular chaperone, HSP90B1 promotes the folding and the assembly of proteins and is induced by an ER stress and the unfolding protein response (UPR) pathway. HSP90B1 is differentially expressed between GC and ABC subtypes of DLBCL and a high HSP90B1 level in ABC-type has an unfavorable prognosis for treated patients [6]. In turn, HSP90B1 overexpression could be associated with chemoresistance. PHB2 (or prohibitin 2) physically interacts with XPO1 [7]. PHB2 is a growth-suppressive protein that has multiple functions both in the nucleus and at the inner mitochondrial membrane [8]. PHB2 together with PHB1 proteins are required for survival of normal lymphocytes [9] and are upregulated in leukemia and lymphoma patients [10]. PPP3CA/B or calcineurin is a calcium-calmodulin-dependent serine/threonine phosphatase that plays a critical role in T-cell activation after TCR engagement. In B cells, a decreased calcineurin activation causes defective B-cell activation [11]. RPN2 (or riphorin II) is a type I integral membrane protein of the ER, and is part of an oligosaccharyltransferase complex that conveys N-linked glycosylation. SCYL2 (or SCYL1-like pseudokinase 2 also known as CVAK104) is a clathrin-associated protein with a serine/threonine activity functioning between the Golgi network and the endosomal system [12]. No relationship between SCYL2 and leukemia/lymphoma or even B-cell has been reported so far. SCYL2/XPO1 interactions have been reported experimentally by affinity capture and MS [13]. TAP1 is a membrane-associated protein, member of the superfamily of ABC transporters involved in multidrug resistance. TAP1 is overexpressed in Hodgkin Reed-Sternberg (HRS) cells of Hodgkin lymphoma (c-HL) [14]. VDAC1 (or voltage-dependent anion channel) is one of the outer mitochondrial membrane component and regulates mitochondria functions *via* the exchanges of metabolites and ions across the membrane. In turn, VDAC1 controls both the metabolic rewiring in tumor cells and mitochondria-mediated apoptosis [15].

3. Supplementary Figures

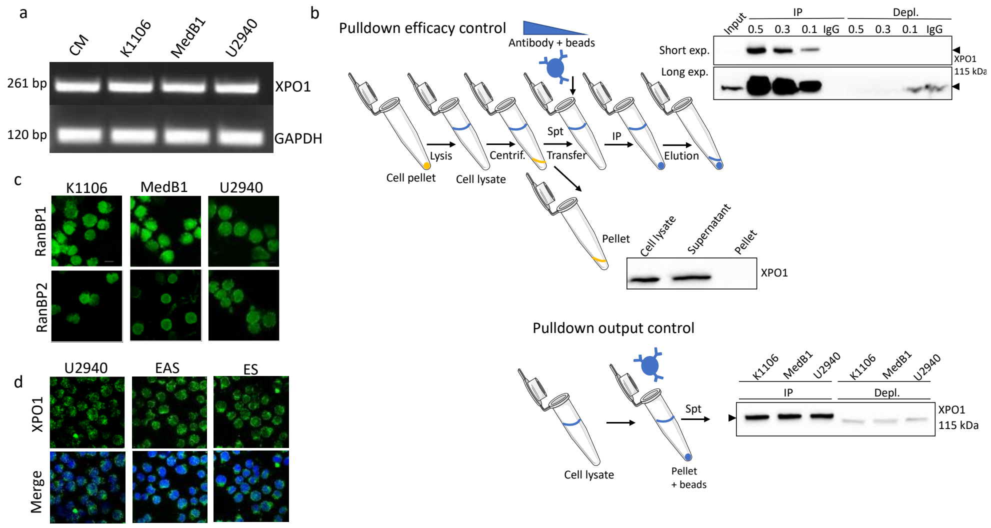

Figure S2. (**a**) Total RNA was purified from the indicated cultured cells, reverse-transcribed and PCR-amplified using primers specific for XPO1 or GAPDH as an internal control (Table S8). PCR products were run on agarose gels (1.8%) and stained with ethidium bromide. Compared to the 50 bp-DNA

ladder, the observed bands were of the expected sizes (on the graph). (**b**) Cultured MedB1 cells were washed three times in PBS and the last supernatant was discarded. Cell pellet was resuspended in the protein extraction buffer containing NP40 and protease inhibitors. The efficiency of extraction was assessed by analysing three fractions at different steps: after cell lysis before centrifugation, after centrifugation within the supernatant and within the pellet. The efficiency of the pulldown was evaluated by incubating the protein suspension with variable amounts of anti-XPO1 Ab (0.5, 0.3 or 0.1 µg). Two µg of IgG were added as a negative control. Immunoprecipitated and depleted (Depl.) proteins were analysed by WB as well as 50 µg of the input as a positive control. For pulldown output control, in the three PMBL cell lines, XPO1 was immunoprecipitated with 0.15 µg of anti-XPO1 Ab for 1 mg of proteins. WB was performed as before with immunoprecipitated and depleted proteins. (**c**) PMBL cells were analysed by IF to determine the localisation of two XPO1 partners. We used primary Abs against RanBP1 and RanBP2 (Table S7) and a goat Alexa Fluor 488-conjugated anti-rabbit IgG as secondary Ab. Slides were counterstained with DAPI and analysed by confocal microscopy (×180, magnification). (**d**) U2640-derived EAS and ES clones having the *XPO1*$^{C528S/E571E}$ allele were analysed by IF to determine the localisation of XPO1. We used an anti-XPO1 Ab (Table S7) and a goat Alexa Fluor 488-conjugated anti-rabbit IgG as secondary Ab. Slides were counterstained with DAPI and analysed by confocal microscopy (×180, magnification).

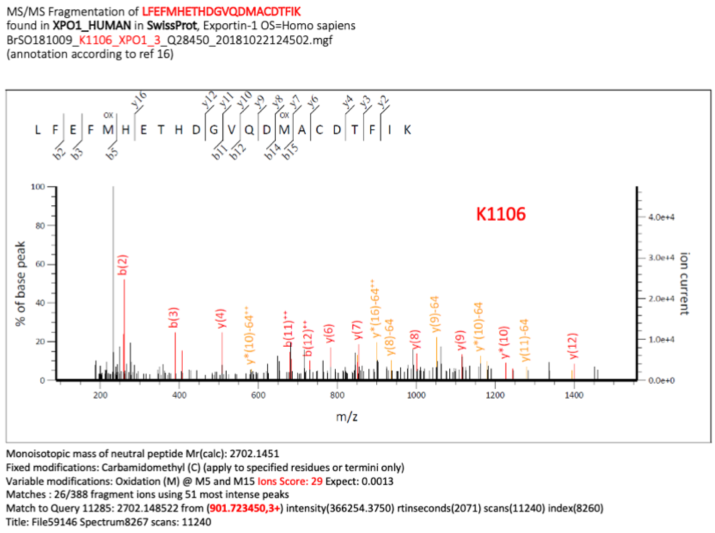

Figure S3. Mascot's database search engine MS/MS annotated spectra [16]. XPO1-derived peptides (zone 568 or 571) from K1106 cells.

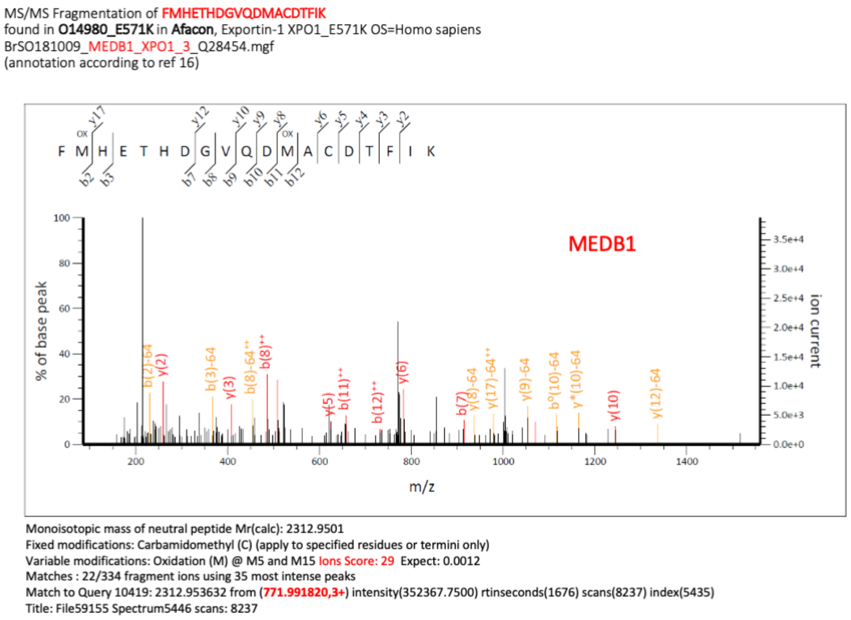

Figure S4. Mascot's database search engine MS/MS annotated spectra [16]. XPO1-derived peptides (zone 568 or 571) from MedB1 cells.

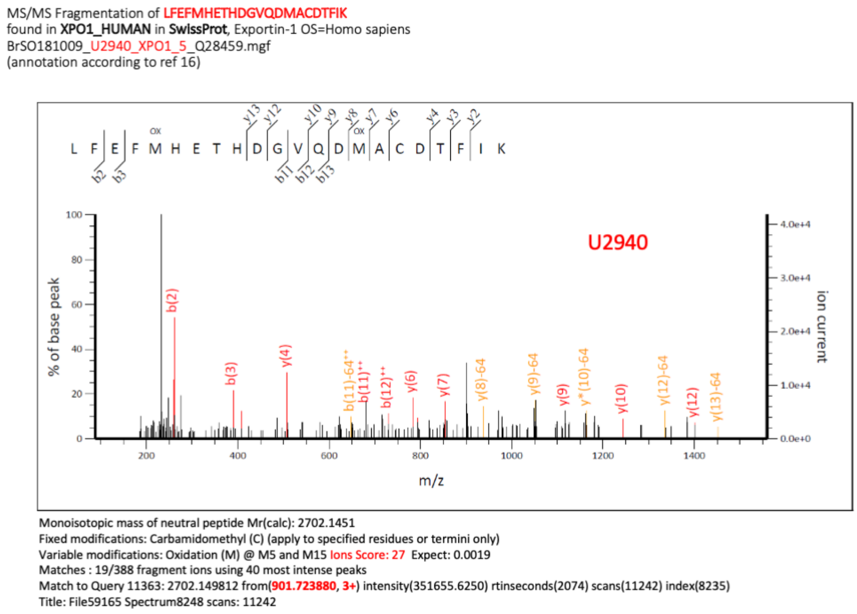

Figure S5. Mascot's database search engine MS/MS annotated spectra [16]. XPO1-derived peptides (zone 568 or 571) from U2940 cells.

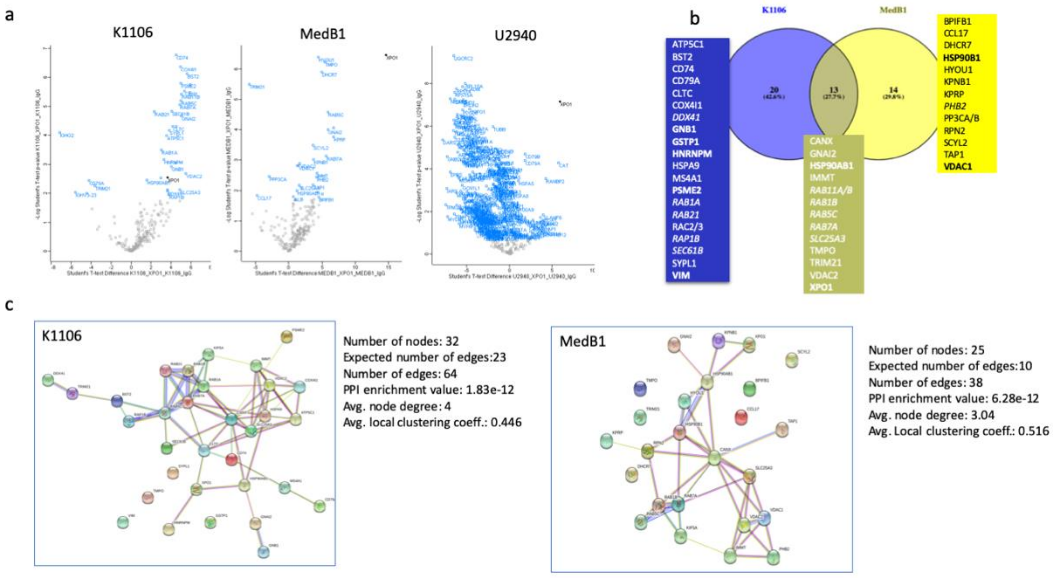

Figure S6. Proteomic analysis of XPO1 interactome in PMBL cells. (**a**) Volcano plots of XPO1 interacting proteins exemplify the characterization of proteins using the IP/MS protocol. Significant interactors are indicated in blue. The XPO1 bait is indicated in black. The volcano plots were generated as previously described [17]; the Y-axis representing the *p*-value and the Y-axis representing the fold change (**b**) The Venn diagram drawn with VENNY software (https://bioinfogp.cnb.csic.es/tools/venny/) shows XPO1 interactors specific to either K1106 or MedB1 or present in both cell lines. In bold are the proteins previously described as XPO1 cargo either by affinity capture or co-fractionation and MS. In italic are the proteins not described as putative cargoes but belonging to a family previously described as a cargo (e.g., DDX41 *vs*. DDX1 or DDX5). Data were extracted from BioGRID v3.5 (https://thebiogrid.org/113348/summary/homo-sapiens/xpo1.html). (**c**) XPO1 networks designed with STRING (v10.5, www.string-db.org) summarise the associations between the characterised 33 proteins (in K1106 cells) and 26 proteins (in MedB1 cells) and XPO1. This analysis predicted a functional enrichment in intracellular transport proteins (GO:0046907).

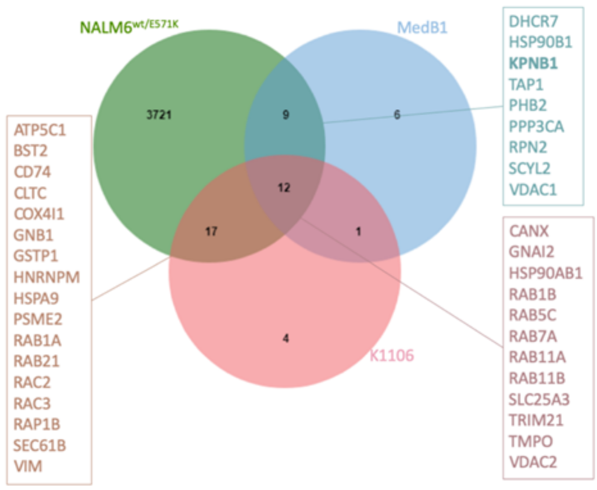

Figure S7. Comparison of XPO1 interactomes in K1106, MedB1 and NALM6 *XPO1*$^{wt/mut}$. The Venn diagram identified proteins common to the three cell lines (in pink) and related to the XPO1^{E571K} mutation (in blue) according to the data of Taylor and coworkers [18] and ours. Among them, KPNB1 (or IPO1, in bold) was further studied.

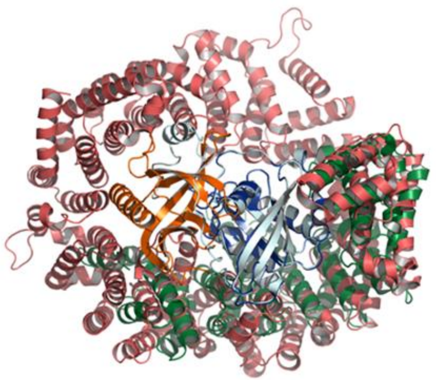

Figure S8. Hypothetical 3D-model of IPO1/XPO1/RanBP2 complex. The model was obtained through the superposition of RanBP2 present in complexes with XPO1 (4GMX) and IPO1 (1IBR) (Protein Data Bank, www.rcsb.org). Each protein is represented in a different colour: XPO1 in red, IPO1 in green, RanGAP in orange, and RanBP2 in blue.

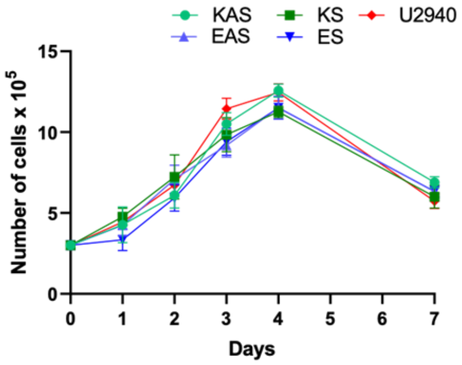

Figure S9. Proliferation curves of U2940 cells, and KS/KAS and EAS/ES derivatives. Cells were seeded at the density of 3 x 10^5 cells/ml in 24-well plates in complete medium and counted by trypan blue exclusion every day for one week (n = 3). During this period, cells were not diluted. The means of total number of living cells are reported on the graph together with SD. The experiment was done two times, a representative one is presented.

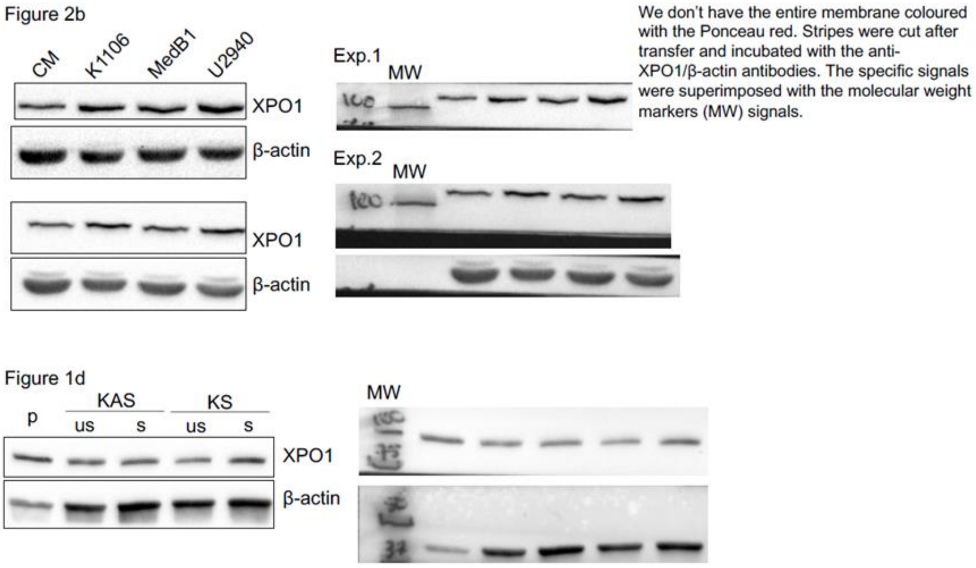

Detail information about Figure 2b.

Figure 4b

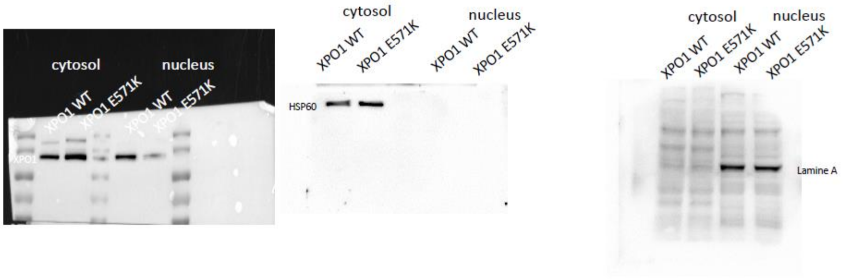

Detail information about Figure 4b.

Figure 5c

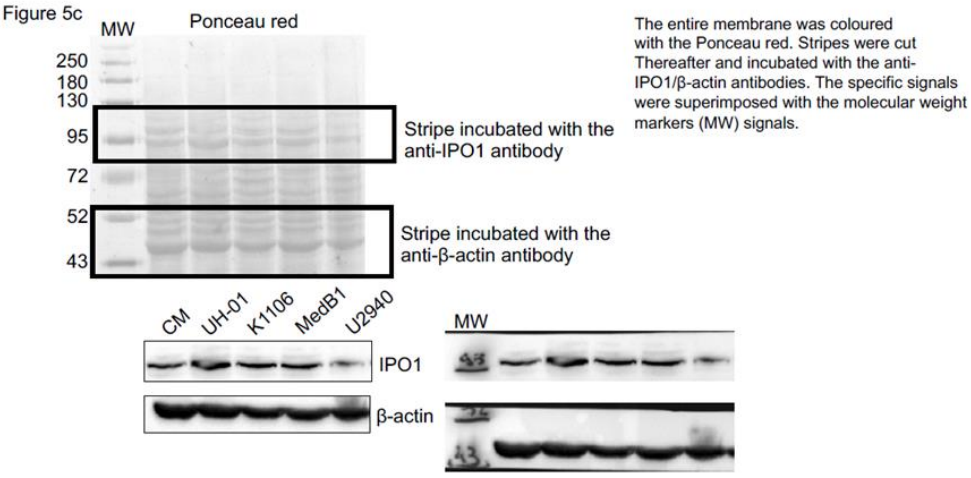

Detail information about Figure 5c.

4. Supplementary Tables

Table S1. Relative quantification of mutant and wild-type (wt) *XPO1* alleles by pyrosequencing.

Cell Line	Material	% of A	% of G
K1106	ADN	7	93
	ARN	9	91
MedB1	ADN	53	47
	ARN	54	46
U2940	ADN	5	95
	ARN	7	93
UH-01	ADN	84	16
	ARN	86	14

gDNA and total RNA were purified from PMBL (K1106, MedB1 and U2940) and cHL (UH-01) cultured cell lines. The exon 15 of *XPO1* was amplified by PCR using the primers listed in Table S2. Pyrosequencing was performed using the XPO1_E571_Seq primer (Table S2). The sequence analysed was the following: 5′-TTC G/AAA TTC ATG-3′.

Table S2. Sequences of the primers used for DNA and RNA pyrosequencing.

Target	Sequences of Primers for Exon 15 Amplification of DNA or RNA
XPO1_F (DNA)	5′-AAA GGT ATG CAA GGG ATA GGT ATG-3′
XPO1_F (RNA)	5′-AGG GAA ACA TTG GTT TAT CTT ACT C-3′
XPO1_R_biot	5′-GAA CCT GAA CGA AAT GCC TG-3′
**Target**	**Sequences of Primers for E571 or C528 Pyrosequencing**
XPO1_E571_Seq	5′-GAA GAC TGT AGT TAA CAA GC-3′
XPO1_C528_Seq	5′-TAC AGG ATC TAT TAG GAT TA-3′

XPO1_F and XPO1_R biotinylated (biot) primers were used for XPO1 exon 15 amplification by PCR (on DNA or RNA). Then pyrosequencing was performed with the Seq primers targeting either E571 or C528 codons. Abreviations: F, forward; R, reverse.

Table S3. XPO1 status in the PMBL and cHL cell lines used in the study.

Cell Line	*XPO1* Status[1]
K1106	wt dupl.
MedB1	wt/E571K
U2940	wt
KAS	wt/C528S + E571K
KS	wt/C528S + E571K
UH-01	wt/E571K dupl.
UH-01Δ	wt/-

[1]Data were obtained from pyrosequencing (Tables S2,S3) and RT-MLPA (not shown) results and from previous published data [19–22]. Abbreviation: dupl., duplicated.

Table S4. Sequences of sgRNA used in the CRISPR-Cas9 studies.

Target	Sequences of sgRNA for Megamer Introduction
XPO1_E571 (sgRNA 1)	5′-AAG CTG TTC **GAA** TTC ATG CA-3′
XPO1_C528 (sgRNA 2)	5′-GGA TTA **TGT** GAA CAG AAA AG-3′
XPO1_K571(sgRNA 3)	5′-AAG CTG TTC **AAA** TTC ATG CA-3′
**Target**	**Sequences of sgRNA for Ultramer Introduction**
XPO1_E571	5′-AAG CTG TTC **GAA** TTC ATG CA-3′
XPO1_K571	5′-AAG CTG TTC **AAA** TTC ATG CA-3′

The bases corresponding to wt (E571) and mutant (K571) human XPO1 gene (locus NM_003400) are in bold and underlined. The same sequences served as crRNA for RNP assembling.

Table S5. Sequences of ssODNs used in the CRISPR-Cas9 barcoding strategies.

Name	Sequences of Megamers
C528S_E571K_S (and _AS)*	5′-GG CTC CAT TAG TGG AGCA ATG CAT GAA GAG GAC GAA AAA CGA TTT CTT GTT ACT GTT ATA AAG GTA TGC AAG GGA TAG GTAT GAA TTA GAA TTG CTA AAT AAG TAT TAT GTT GTT ACA ATA AAT AAT ACA AAT TTG TCT TAT TTA CAG GAT CTA TTA GGA TTA **TCA** GAA CAG AAg cGA GGC AAA GAT AAT AAA GCT ATT ATT GCA TCA AAT ATC ATG TAC ATA GTA GGT CAA TAC CCA CGT TTT TTG AGA GCT CAC TGG AAA TTT CTG AAG ACT GTA GTT AAC AAG CTt TTC **GA**g TTt ATG CAT GGT AAA TCT CTT TCT TTA CTA TAT TTT GCT TTT ATT TTT ATT GAA GAA AAT AAA TGA ATG TTT TTG TCT TGT TAG AGA CCC ATG ATG GAG TCC AGG-3′
C528S_E571E_S (and _AS)*	5′-GG CTC CAT TAG TGG AGCA ATG CAT GAA GAG GAC GAA AAA CGA TTT CTT GTT ACT GTT ATA AAG GTA TGC AAG GGA TAG GTAT GAA TTA GAA TTG CTA AAT AAG TAT TAT GTT GTT ACA ATA AAT AAT ACA AAT TTG TCT TAT TTA CAG GAT CTA TTA GGA TTA **TCA** GAA CAG AAg cGA GGC AAA GAT AAT AAA GCT ATT ATT GCA TCA AAT ATC ATG TAC ATAGTA GGT CAA TAC CCA CGT TTT TTG AGA GCT CAC TGG AAA TTT CTG AAG ACT GTA GTT AAC AAG CTt TTC **GA**g TTt ATG CAT GGT AAA TCT CTT TCT TTA CTA TAT TTT GCT TTT ATT TTT ATT GAA GAA AAT AAA TGA ATG TTT TTG TCT TGT TAG AGA CCC ATG ATG GAG TCC AGG-3
**Name**	**Sequences of Ultramers**

ssXPO1_E571E	5′-GAT AAT AAA GCT ATT ATT GCA TCA AAT ATC ATG TAC ATA GTA GGT CAA TAC CCA CGT TTc TTG AGA GCT CAC TGG AAA TTT CTG AAt ACg GTg GTc AAt AAG CTc TTC **GAg** TTt ATG CAT GGT AAA TCT CTT TCT TTA CTA TAT TTT GCT TTT ATT TTT ATT GAA GAA AAT AAA TGA ATG TTT TTG TCT TG-3′
ssXPO1_E571K	5′-GAT AAT AAA GCT ATT ATT GCA TCA AAT ATC ATG TAC ATA GTA GGT CAA TAC CCA CGT TTc TTG AGA GCT CAC TGG AAA TTT CTG AAG ACc GTc GTg AAC AAa CTc TTt **aAg** TTt ATG CAT GGT AAA TCT CTT TCT TTA CTA TAT TTT GCT TTT ATT TTT ATT GAA GAA AAT AAA TGA ATG TTT TTG TCT TG-3′

Codons to be targeted are in bold. The PAM sequences are underlined. Silent mutations introduced in the barcodes are in red and in lower case. They were designed according to Guernet et al. [1] *The sequence of ssODN C528S_E571K_AS and C528S_E571E_AS are the reverse.

Table S6. Selinexor treatment allows the recovery of U2940 cells having the the two C528S/E571K mutations.

		No Selinexor Treatment			With Selinexor Treatment		
Cells	Position	1	2	3	1	2	3
KAS	A		9			45	
	C			70			16
	G	88	91		37	55	
	T	12		30	63		84
KS	A		28			47	
	C			19			18
	G	70	72		58	53	
	T	30		81	42		82
EAS	A		60			3	
	C			61			8
	G	72	40		9	97	
	T	28		39	91		92
ES	A		20			10	
	C			21			10
	G	26	80		20	90	
	T	74		79	80		90

U2940 cells were transfected with sense or antisense (S and AS) megamers for introducing the two C528S and E571K mutations (C528S_E571K_S and AS, KS and KAS, Table S5) or the C528S mutation and E571E substitution (C528S_E571E_S and AS, ES and EAS, Table S5). Two weeks after the nucleofection, cells were treated (or not for a control) with selinexor (1 µM) for one week then analysed by pyrosequencing. The sequences of interest are the following: CTG TTC GAA TTC ATG, wt sequence, with the changing bases underlined and the targeted codon highlighted; CTT TTC AAA TTT ATG, double mutant sequence, with the mutant bases in red and underlined, corresponding to the positions 1, 2 and 3 in the table. The percentage of each base at each position obtained by pyrosequencing is indicated. We observed an increase of the percentage of the expected base at the correct position upon selinexor treatment.

Table S7. Primary antibodies used in the study.

Target	Reference	Origin	Assay (Dilution of Ab)
HSP60	#12165	Cell Signaling Technology	WB (1/1000)
IPO1	HPA050302	Sigma-Aldrich	IF (1/1000)<br>PLA (1/1000)
Lamin A/C	#4777	Cell Signaling Technology	WB (1/1000)
NPM	ab10530	abcam	IF (1/500)
RanBP1	ab97659	abcam	IF (1/50)
RanBP2	ab64276	abcam	IF (1/50)<br>PLA (1/50)
RELA	HPA063451<br>ab32436	Sigma-Aldrich<br>abcam	IF (1/200)<br>IF (1/50)
XPO1	sc-74454<br>A300-469A	Santa Cruz<br>Bethyl	WB (1/500)<br>IF (1/500)<br>PLA (1/500)

Abbreviations: IF, immunofluorescence; PLA, proximity ligation assay, and WB, western blotting.

Table S8. Sequences of the primers used for RT-PCR and Sanger sequencing.

Gene	Sequence
XPO1	F, 5′-GAG CTC ACT GGA AAT TTC TGA AGA C-3′<br>R, 5′-CCA ATC ATG TAC CCC ACA GCT TC-3′
GAPDH	F, 5′-CTG ACT TCA ACA GCG ACA CCC-3′<br>R, 5′-CCC TGT TGC TCT AGC CAA AT-3′

Abbreviations: F, forward; R, reverse. The same primers were designed for PCR amplification of *XPO1* and PCR fragment Sanger sequencing. Primer sequences were designed with the primer 3 software (v4.0, //primer3.ut.ee/).

Table S9. Proximity-ligation assay results.

Cell Line	MedB1	UH-01	U2940
Nb. of cells	223	148	238
	167	150	248
	192	141	278
Nb. of spots	250	82	97
	106	102	115
	120	110	59
Nb. of spots/100 cells	67	55	41
	63	68	46
	62	78	22

The Duolink PLA technology was used on MedB1, UH-01 and U2940 cell lines to investigate XPO1/IPO1 interactions. Slides were incubated with the primary Abs (Table S7), except for the negative control, and with the secondary Abs conjugated with the PLUS and MINUS probes. The ligation and amplification steps were next performed and the slides were counterstained with DAPI and observed with a confocal microscope (×180, magnification). The number of red dots was counted on at least 70 cells on one slide. For each experiment two slides were set up, and three independent experiment have been done. The number of cells with at least one red dot as well as the number of red dots were counted as indicated.

Table S10. Sequences of the primers used for PCR in CRISPR-Cas9 assays.

Target	Sequence
XPO1ext	F, 5′-TGT GTT GGG CAA TAG GCT CC-3′<br>R, 5′-TTC ATA CCT ATC CCT TGC ATA CCT T-3′
XPO1_E571	F, 5-AGG ATT ATG TGA ACA GAA AAG AGG C-3′
XPO1_E571K	R, 5′-CAT AAA CTT AAA GAG TTT GTT CAC GAC G-3′
XPO1_E571E	R, 5′-AGAGCTTATTGA CCA CCG TAT TC-3′

Abbreviations: F, forward; R, reverse. Primer sequences for PCR amplification were designed with the primer 3 software (v4.0, //primer3.ut.ee/). To quantify the proportion of edited cells, we used reverse primers specific for the E571K (XPO1_E571K) or the E571E (XPO1_E571E) barcodes. The same forward primer was used. For normalization, we used XPO1ext primers which are distant from the edited region.

5. Supplementary References

1. Guernet, A.; Mungamuri, S.K.; Cartier, D.; Sachidanandam, R.; Jayaprakash, A.; Adriouch, S.; Vezain, M.; Charbonnier, F.; Rohkin, G.; Coutant, S.; et al. CRISPR-barcoding for intratumor genetic heterogeneity modeling and functional analysis of oncogenic driver mutations. *Mol. Cell* **2016**, *63*, 526–538.
2. Ran, F.A.; Hsu, P.D.; Wright, J.; Agarwala, V.; Scott, D.A.; Zhang, F. Genome engineering using the CRISPR-Cas9 system. *Nat. Protoc*. **2013**, *8*, 2281–308.

3. Ritz, O.; Guiter, C.; Castellano, F.; Dorsch, K.; Melzner, J.; Jais, J.P. Recurrent mutations of the STAT6 DNA binding domain in primary mediastinal B-cell lymphoma. *Blood* **2009**, *114*, 1236–1242.
4. Kulling, P.M.; Olson, K.C.; Olson, T.L.; Feith, D.J.; Loughran Jr., T.P. Vitamin D in haematological disorders and malignancies. *Eur. J. Haematol*. **2017**, *98*, 187–197.
5. Falsone, S.F.; Gesslbauer, B.; Tirk, F.; Piccinini, A.M.; Kungl, A.J. A proteomic snapshot of the human heat shock protein 90 interactome. *FEBS Lett*. **2005**, *579*, 6350–6354.
6. Boelens, J.; Jais, J.P.; Vanhoecke, B.; Beck, I.; Van Melckebeke, H.; Philippe, J.; Bracke, M.; Jardin, F.; Briere, J.; Leroy, K.; et al. ER stress in diffuse large B cell lymphoma: GRP94 is a possible biomarker in germinal versus activated B-cell type. *Leuk. Res*. **2013**, *37*, 3–8.
7. Rastogi, S.; Joshi, B.; Fusaro, G.; Chellappan, S. Camptothecin induces nuclear export of prohibitin preferentially in transformed cells through a CRM1-dependent mechanism. *J. Biol. Chem*. **2006**, *281*, 2951–2959.
8. Zhou, Z.; Ai, H.; Li, K.; Yao, X.; Zhu, W.; Liu, L.; Yu, C.; Song, Z.; Bao, Y.; Huang, Y.; et al. Prohibitin 2 localizes in nucleolus to regulate ribosomal RNA transcription and facilitate cell proliferation in RD cells. *Sci. Rep*. **2018**, *8*, 1479.
9. Ross, J.A.; Nagy, Z.S.; Kirken, R.A. The PHB1/2 phosphocomplex is required for mitochondrial homeostasis and survival of human T cells. *J. Biol. Chem*. **2008**, *283*, 4699–4713.
10. Ross, J.A.; Robles-Escajeda, E.; Oaxaca, D.M.; Padilla, D.L.; Kirken, R.A. The prohibitin protein complex promotes mitochondrial stabilization and cell survival in hematologic malignancies. *Oncotarget* **2017**, *8*, 65445–65456.
11. Bhattacharyya, S.; Deb, J.; Patra, A.K.; Thuy Pham, D.A.; Chen, W.; Vaeth, M.; Berberich-Siebelt, F.; Klein-Hessling, S.; Lamperti, E.D.; Reifenberg, K.; et al. NFATc1 affects mouse splenic B cell function by controlling the calcineurin-NFAT signaling network. *J. Exp. Med*. **2011**, *208*, 823–839.
12. Duwel, M.; Ungewickell, E.J. Clathrin-dependent association of CVAK104 with endosomes and the trans-Golgi network. *Mol. Biol. Cell* **2006**, *17*, 4513–4525.
13. Kırlı, K.; Karaca, S.; Dehne, H.J.; Samwer, M.; Pan, K.T.; Lenz, C.; Urlaub, H.; Goerlich, D. A deep proteomics perspective on CRM1-mediated nuclear export and nucleocytoplasmic partitioning. *eLife* **2015**, *4*, e11466, doi:10.7554/eLife.11466
14. Murray, P.G.; Constandinou, C.M.; Crocker, J.; Young, L.S.; Ambinder, R.F. Analysis of major histocompatibility complex class I, TAP expression, and LMP2 epitope sequence in Epstein-Barr virus-positive Hodgkin's disease. *Blood* **1998**, *92*, 2477–2483.
15. Shoshan-Barmatz, V.; Maldonado, E.N.; Krelin, Y. VDAC1 at the crossroads of cell metabolism, apoptosis and cell stress. *Cell Stress* **2017**, *1*, 11–36.
16. Perkins, D.N.; Pappin, D.J.; Creasy, D.M.; Cottrell, J.S. Probability-based protein identification by searching sequence databases using mass spectrometry data. *Electrophoresis* **1999**, *20*, 3551–3567.
17. Giai Gianetto, Q.; Coute, Y.; Bruley, C.; Burger, T. Uses and misuses of the fudge factor in quantitative discovery proteomics. *Proteomics* **2016**, *16*, 1955–1960.
18. Taylor, J.; Sendino, M.; Gorelick, A.N.; Pastore, A.; Chang, M.T.; Penson, A.V.; Gavrila, E.I.; Stewart, C.; Melnik, E.M.; Herrejon Chavez, F.; et al. Altered nuclear export signal recognition as a driver of oncogenesis. *Cancer Discov*. **2019**, *9*, 1452–1467.
19. Camus, V.; Stamatoullas, A.; Mareschal, S.; Viailly, P.J.; Sarafan-Vasseur, N.; Bohers, E.; Dubois, S.; Picquenot, J.M.; Ruminy, P.; Maingonnat, C.; et al. Detection and prognostic value of recurrent exportin 1 mutations in tumor and cell-free circulating DNA of patients with classical Hodgkin lymphoma. *Haematologica* **2016**, *101*, 1094–1101.
20. Dai, H.; Ehrentraut, S.; Nagel, S.; Eberth, S.; Pommerenke, C.; Dirks, W.G.; Geffers, R.; Kalavalapalli, S.; Kaufmann, M.; Meyer, C.; et al. Genomic landscape of primary mediastinal B-cell lymphoma cell lines. *PLoS ONE* **2015**, *10*, e0139663.
21. Quentmeier, H.; Pommerenke, C.; Dirks, W.G.; Eberth, S.; Koeppel, M.; MacLeod, E.A.F.; Nagel, S.; Steube, K.; Uphoff, C.C.; Drexler, H.G. The LL-100 panel: 100 cell lines for blood cancer studies. *Sci. Rep*. **2019**, *9*, 8218.
22. Liu, Y.; Abdul Razak, F.R.; Terpstra, M.; Chan, F.C.; Saber, A.; Nijland, M.; van Imhoff, G.; Visser, L.; Gascoyne, R.; Steidl, C.; et al. The mutational landscape of Hodgkin lymphoma cell lines determined by whole-exome sequencing. *Leukemia* **2014**, *28*, 2248–2251.

© 2020 by the authors. Licensee MDPI, Basel, Switzerland. This article is an open access article distributed under the terms and conditions of the Creative Commons Attribution (CC BY) license (http://creativecommons.org/licenses/by/4.0/).
